# Establishment of Z Score Reference of Growth Parameters for Egyptian School Children and Adolescents Aged From 5 to 19 Years: A Cross Sectional Study

**DOI:** 10.3389/fped.2020.00368

**Published:** 2020-07-21

**Authors:** Ali M. El Shafie, Fady M. El-Gendy, Dalia M. Allahony, Zein A. Omar, Mohamed A. Samir, Ahmed N. El-Bazzar, Mohamed A. Abd El-Fattah, Amir A. Abdel Monsef, Amir M. Kairallah, Hythem M. Raafet, Ghada M. Baza, Amany G. Salah, Walaa S. Galab, Zeinab A. Kasemy, Wael A. Bahbah

**Affiliations:** ^1^Department of Pediatrics, Faculty of Medicine, Menoufia University, Shebeen El-Kom, Egypt; ^2^Ministry of Health Hospitals, Cairo, Egypt; ^3^Department of Public Health and Community Medicine, Faculty of Medicine, Menoufia University, Shebeen El-Kom, Egypt

**Keywords:** adolescent, Egyptian Z score, growth parameters, nutritional status, school children

## Abstract

**Background:** Growth charts are an important method for evaluating a child's health, growth, and nutritional status.

**Objective:** To establish Lambda—Mu- Sigma (LMS) and Z score references for assessment of growth and nutritional status in Egyptian school children and adolescents.

**Methods:** A total of 34,822 Egyptian school children and adolescents from 5 to 19 years were enrolled in a cross sectional randomized study from December 2017 to November 2019 to create LMS and Z score references for weight, height and body mass index (BMI) corresponding to ages. They were selected from different districts in Egypt. Apparent Healthy children with good nutritional history and not suffering from any chronic diseases were included in the study.

**Results:** Egyptian children of both sexes (54.3% boys and 45.7 % girls) from 5 to 19 years old were studied. Then LMS and Z scores for weight for age, height for age, BMI for age of both sexes were represented in detailed tables and graphs. There was no statistically significant difference between the Egyptian Z score charts and the reference values of WHO for weight, height and BMI corresponding to age (*P* > 0.05).

**Conclusion:** This is the first national reference for growth and nutritional assessment using LMS and Z score charts in Egyptian school children and adolescents, this tool is essential for healthcare and research.

## Introduction

Impairment in growth is one of universal public health problems (1), and its early detection and accurate diagnosis is important for early intervention (2). Age- and sex-specific growth charts are the important tools used for monitoring children's longitudinal growth (3–5).

For investigating a child's growth rate, weight and height are the most common anthropometric measurements used. So assessment of weight and height plays an important role in follow up of the growth and nutritional status and early detection of its abnormalities for early intervention before permanent changes occur (6).

Over the past decades, one of the most important health problem worldwide is the increasing pattern of childhood obesity (7–11). Beyond the research setting, Body mass index is used alone as indicator of overweight and obesity in children and adolescents (12, 13).

In Egypt two thirds of child mortality owed to malnutrition and stands as one of the 36 countries where 90 % of the global burden of malnutrition falls (14). Pediatric overweight and obesity are considered the most prevalent nutritional disorder among both children and adolescents with 21–24% of them are overweight (15).

The first national charts on the physical Growth and development of children were established in 1972 (16, 17). This study was conducted on 2,121 Egyptian children from Cairo area aged from 6 to 18 years and measured weight, stature and weight/stature index using percentiles method.

The second national charts were done in 2002 using the same study design and in the same district but it included more age group (from birth to 18 years) (18).

In Egypt, there are two international references charts used to assess growth and nutritional status of children and adolescents aged 5–19 years, the first one is the recently updated World Health Organization (WHO) 2007 (19). while the second is the reference values from the Centers for Disease Control and Prevention (CDC) (20). Egypt does not have its own growth reference data based on Lambda—Mu- Sigma (LMS) and Z score parameters that is constructed with a representative national sample of children and adolescents, so the aim of this study was to establish a national reference charts for assessment of growth and nutritional status in Egyptian school children and adolescents by using LMS and Z score parameters.

## Methods and Design

### Participants

Egypt consists of 27 governorates. Cairo is the largest governorates in terms of population, accounting for one-third of Egypt's population according to the latest statistics. Therefore, this was taken into account and the largest number of participants from Cairo. To conduct the study, a cross-sectional design with a multistage random sampling technique was used. Out of 27 governorates, three governorates from Upper Egypt, and five governorates including Cairo from Lower Egypt were chosen in this study. We chose 18 districts from previously chosen governorates randomly. Then facilities including basic education schools and secondary training schools were counted, and 140 facilities in the selected 8 governorates were randomly chosen. The study sample was determined based on Egypt demographic health survey 2015 (21). All the socioeconomic strata were represented with weighted rural-urban representation. All details of the selected households in cities and villages in every single governorate were provided. Any child aged from 5 to 19 years old was eligible to share in the study. The process was totally computerized. The involved team (primary care physicians, pediatricians, and nurses) had been subjected to a training workshop on anthropometric measurements over 2 days followed by testing to avoid inter and intra-observer bias.

Out of the week, the study was conducted in three crowded days to gather more information. They were selected from Primary health centers, Primary school and secondary school as representative of children in Egypt. Between the educational courses and during break time, children were screened in the school that was selected randomly from the list of schools.

A total of 34,822 Egyptian children from 5 to 19 years were studied from December 2017 to November 2019. Firstly, we took 35,042 who were eligible for the study, then 34,822 children and adolescents were selected as a final total sample after exclusion of 220 children on applying the inclusion and exclusion criteria.

### Ethics Approval

Institutional Review Boards (IRB) of the Menoufia faculty of medicine had approved the study. Ethical approval ID is 171112Ped. Research work had been performed in accordance with the Declaration of Helsinki. Purpose of the study was explained to all mothers/guardians of the children and adolescents and informed about objective of the study with absence of any risk to their children in participation in this study and those who agreed to participate signed an informed consent to confirm their willingness to participate.

### Inclusion and Exclusion Criteria

All apparent healthy children with good developmental and nutritional history were included in this study. We excluded any child suffering from any chronic disease (cardiac, hematological, renal, endocrinal and hepatic diseases), fever or documented underlying disease at the time of examination.

### Measurements and Data Collection

We measured weight for age, height for age, and calculate BMI for age for both sexes. All measured were collected by trained medical staff. All children were examined by identical measuring equipment. Weight was measured by a digital balanced scale (Beurer model GS 11, Germany) and height assessed by Harpenden fixed stadiometer. Device was calibrated daily. BMI was calculated using the formula: BMI = weight (Kg)/[height (m)]^2^.

### Patient and Public Involvement

A sample of 99 children and caregivers beside 81 healthcare providers were interviewed and invited to provide us with their background questions related to anthropometric measurements. Four top questions out of 120 related ones were highlighted and studied in the attached figures. The aim of this step was to enlighten the way to our research to be relevant to the health needs.

### Statistical Analysis

The LMS parameters were used to determine the standard deviation (−3 to +3) of weight, height and BMI for both sexes. The median (M), the generalized coefficient of variation (S), and the power in the Box–Cox transformation (L) are the content of LMS parameters. To convert the distribution of data to normal distribution, we used method of The Box–Cox transformation. The method models the data taking into consideration the degree of skewness (L), central tendency (M), and dispersion (S). The L, M, and S parameters are calculated and smoothed according to the method of maximum penalized likelihood (22, 23). Z score and LMS parameters were calculated by using the Statistical package SPSS, version 20, for windows (SPSS Inc., Chicago, Illinois, USA) and Excel according to the following formula:

$$\begin{array}{l} \text{P}=\text{M}\left[1+\text{LSZ}\right]1/\text{L},\text{L}\neq \;0 \end{array}$$

We calculated the z-score values of −3, −2, −1, 0, +1, +2, and +3 for weight, height and BMI for age. The goodness of fit of all L, M, and S models was assessed using Q-test (24).

Student *t*-test for one sample was used to compare the means values of each group variable with the Egyptian and WHO reference values. For all analyses, the significance level was set at 5%.

## Results

A total of 34,822 Egyptian children (54.3% boys and 45.7 % girls), from 5 to 19 years of age (Table 1)

**Table 1:** Number of participating children in each age group/year.

	**Boy**	**Girl**
5 years	1,199	1,135
6 years	1,259	1,096
7 years	1,288	1,082
8 years	1,290	1,050
9 years	1,244	1,026
10 years	1,283	1,088
11 years	1,305	1,030
12 years	1,254	1,047
13 years	1,284	1,063
14 years	1,247	1,053
15 years	1,299	1,036
16 years	1,261	1,049
17 years	1,248	1,073
18 years	1,210	1,034
19 years	1,243	1,046
Total	18,914	15,908

**Figure 1:**
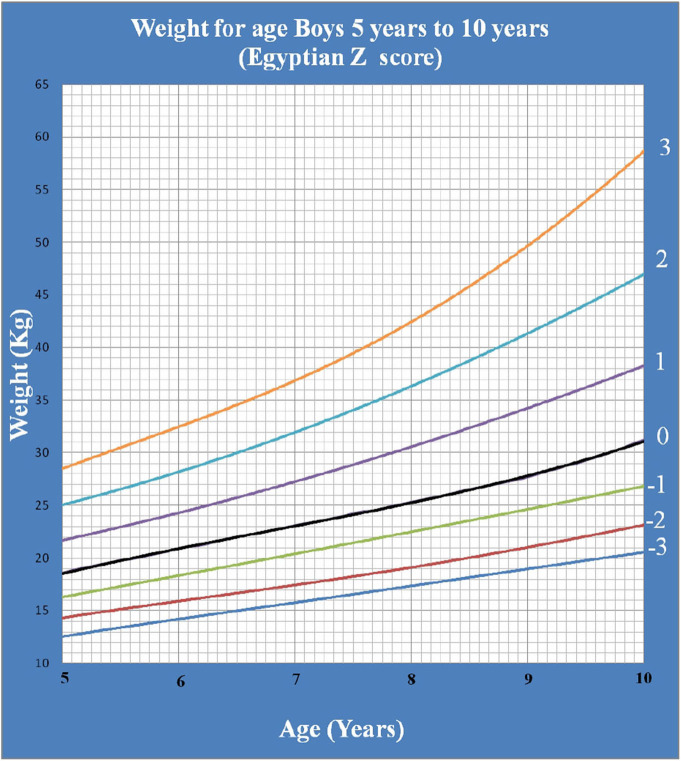
The Egyptian Z score weight for age from 5 years to 10 years for boys.

**Figure 2:**
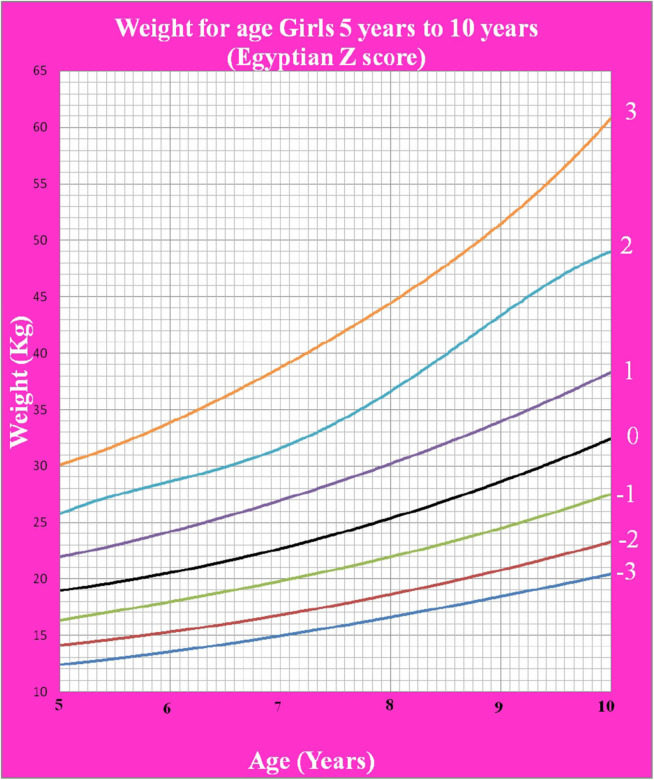
The Egyptian Z score weight for age from 5 years to 10 years for girls.

**Figure 3:**
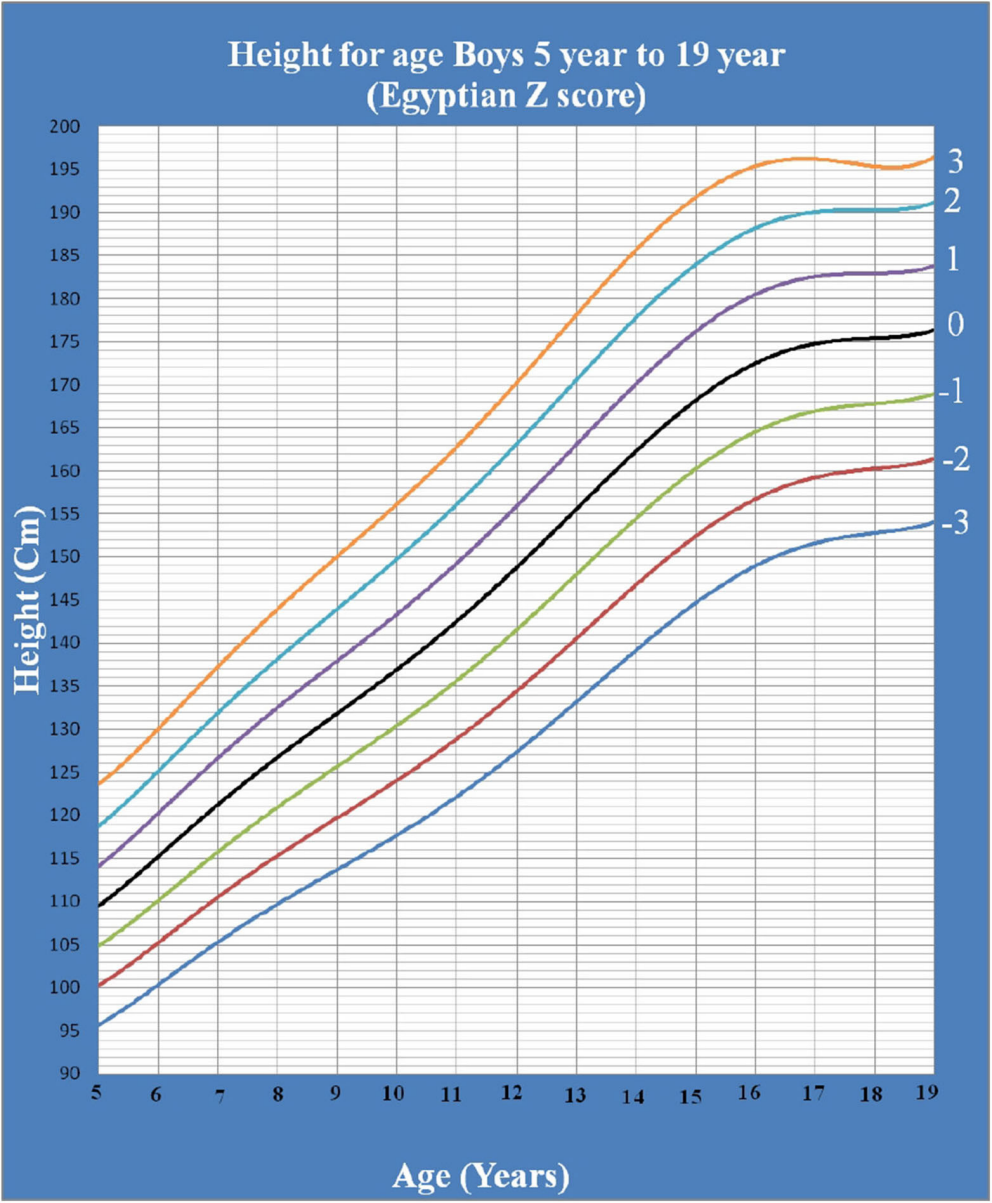
The Egyptian Z score height for age from 5 years to 19 years for boys.

**Figure 4:**
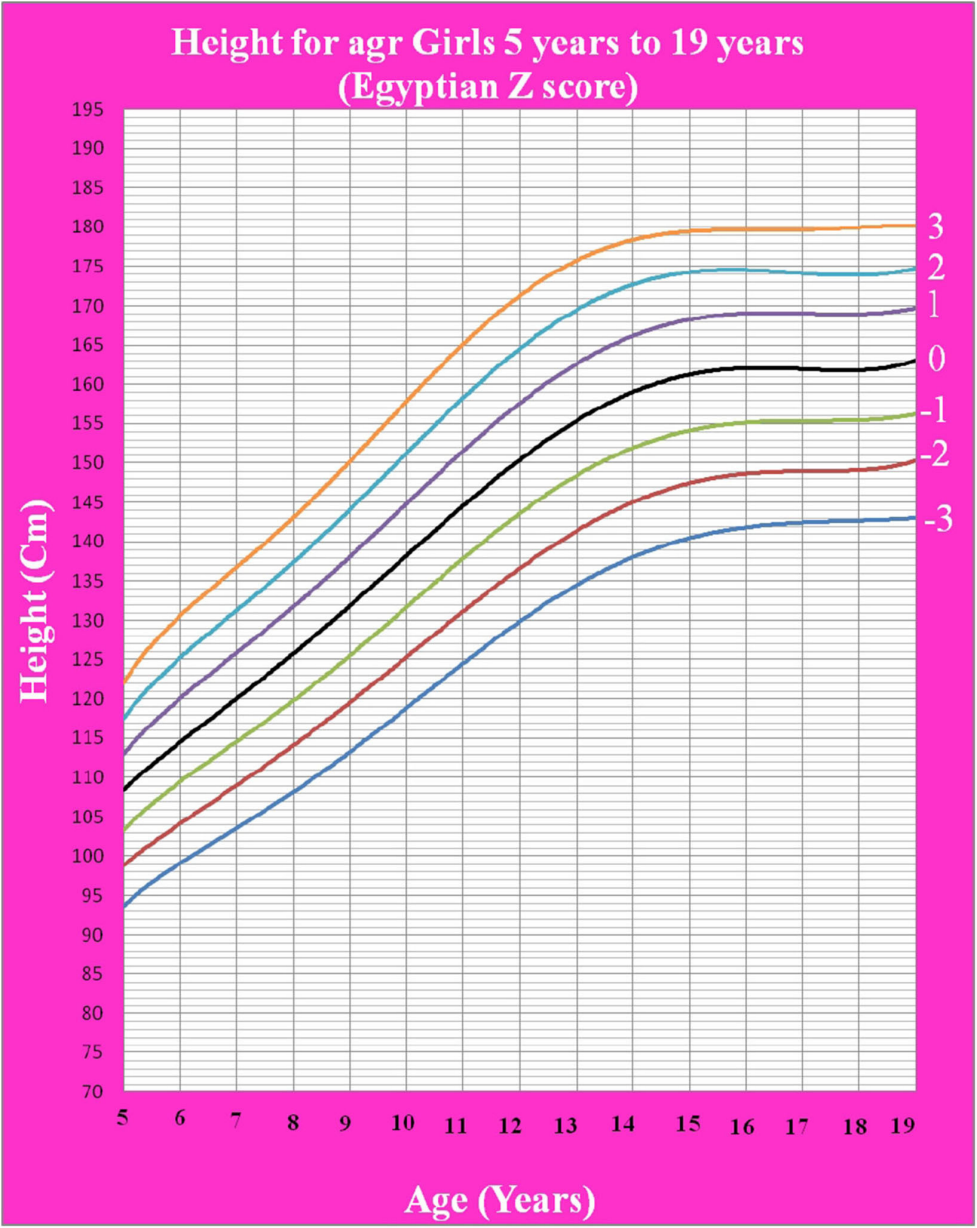
The Egyptian Z score height for age from 5 years to 19 years for girls.

**Figure 5:**
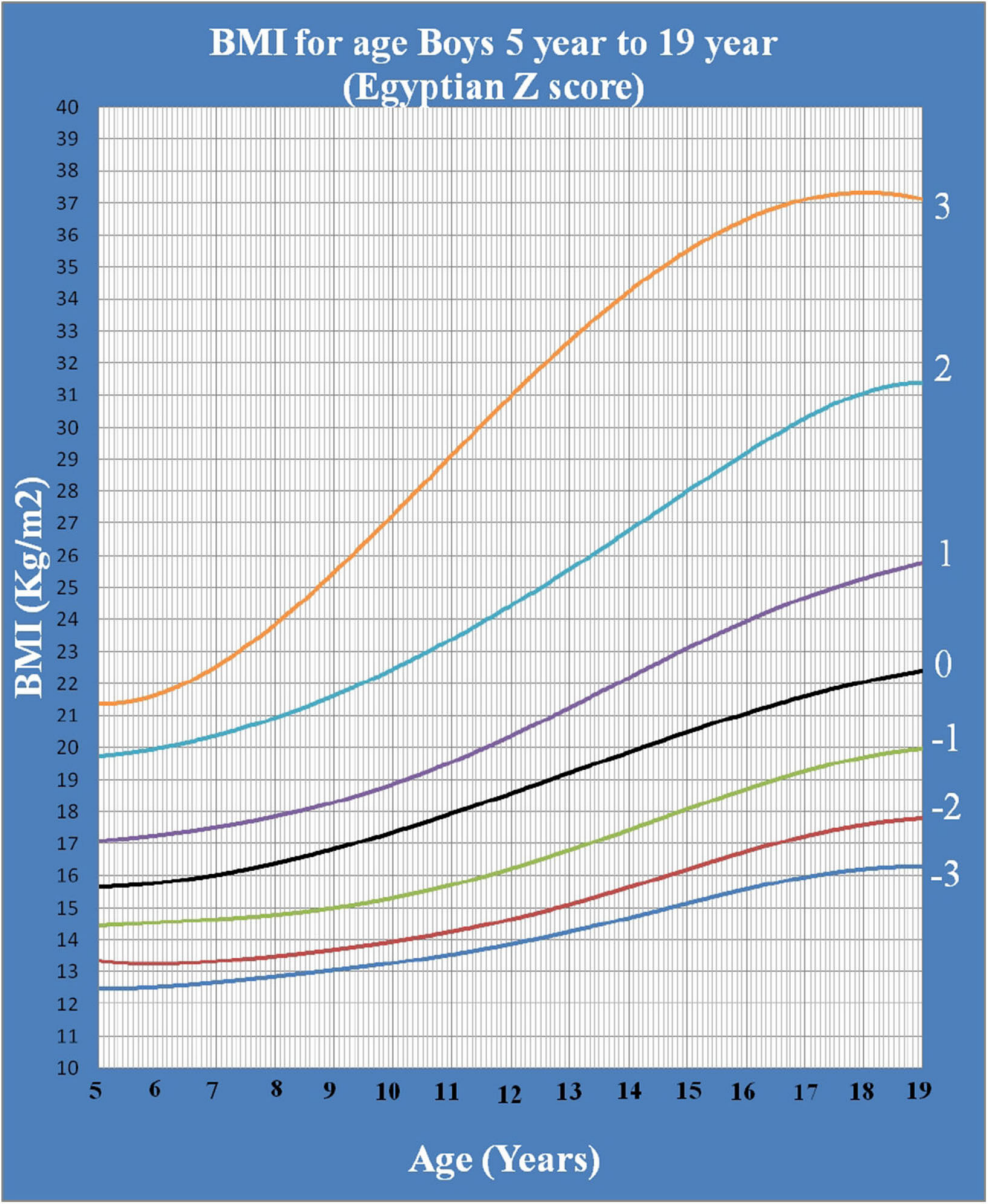
The Egyptian Z score BMI for age from 5 year to 19 years for boys.

**Figure 6:**
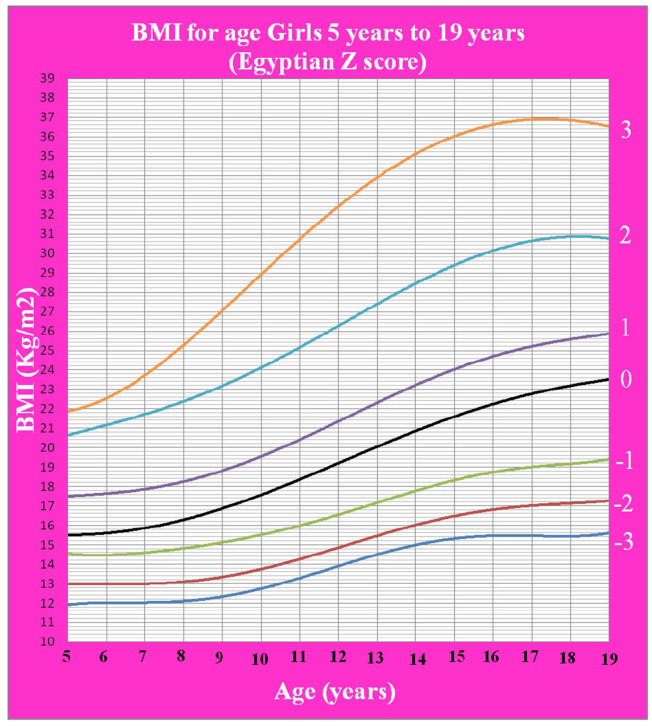
The Egyptian Z score BMI for age from 5 years to 19 years for girls.

**Figure 7:**
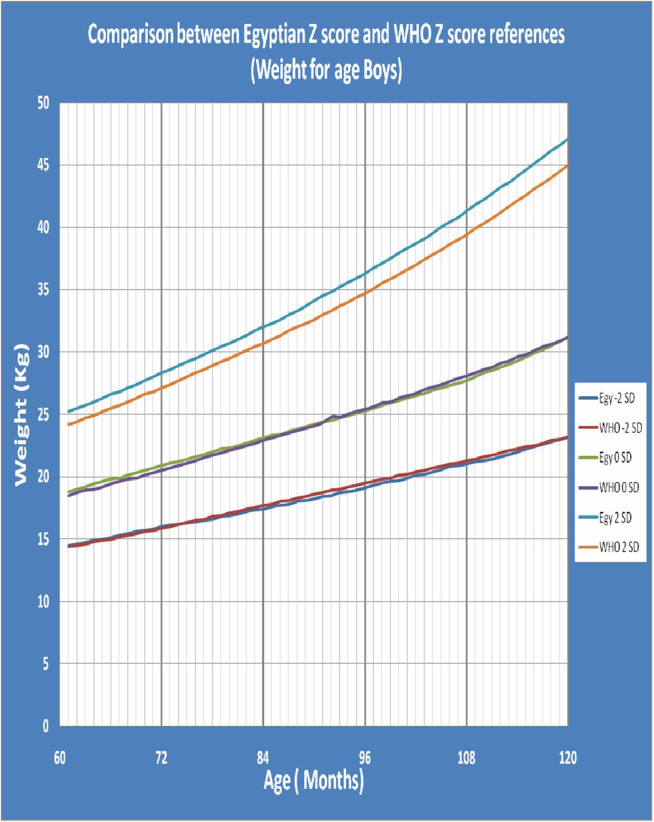
The comparison between Egyptian Z score and WHO Z score references value (weight for age in boys).

**Figure 8:**
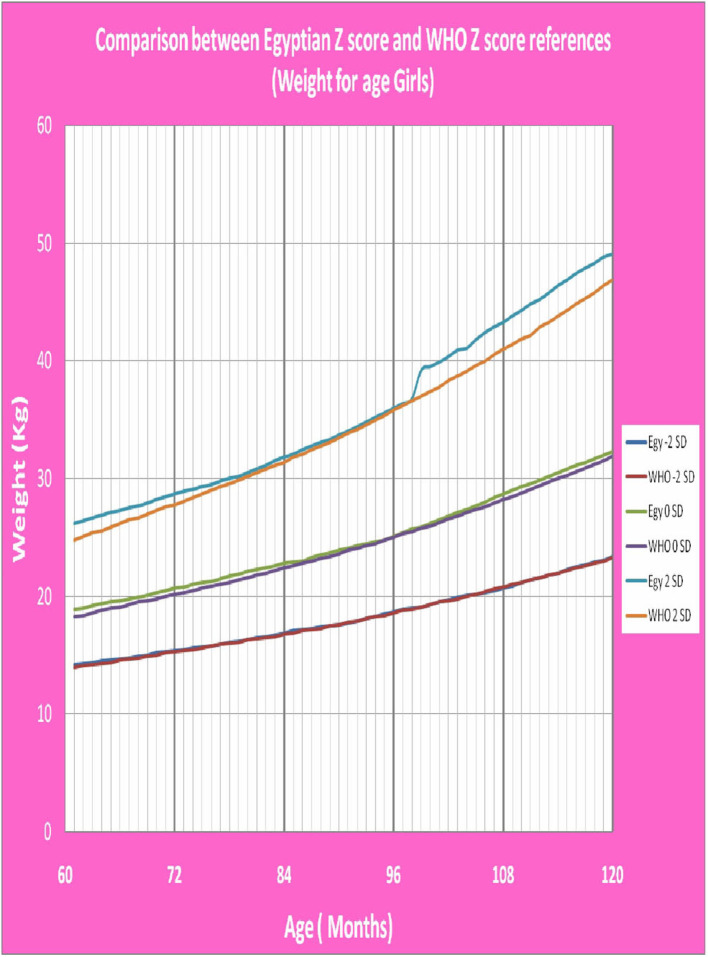
The comparison between Egyptian Z score and WHO Z score references value (weight for age in girls).

**Figure 9:**
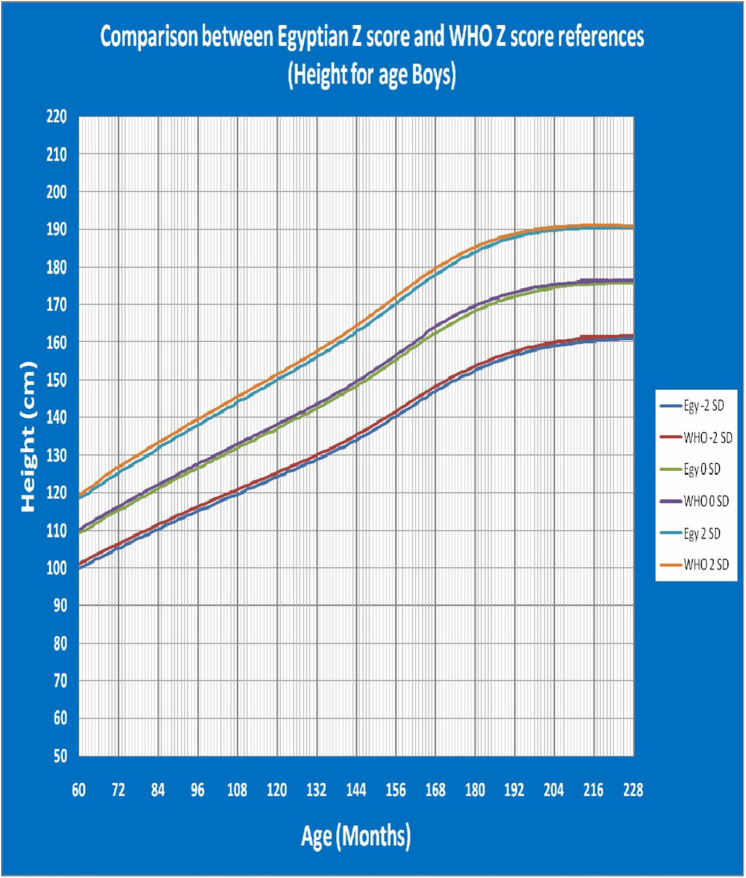
The comparison between Egyptian Z score and WHO Z score references value (Height for age in boys).

**Figure 10:**
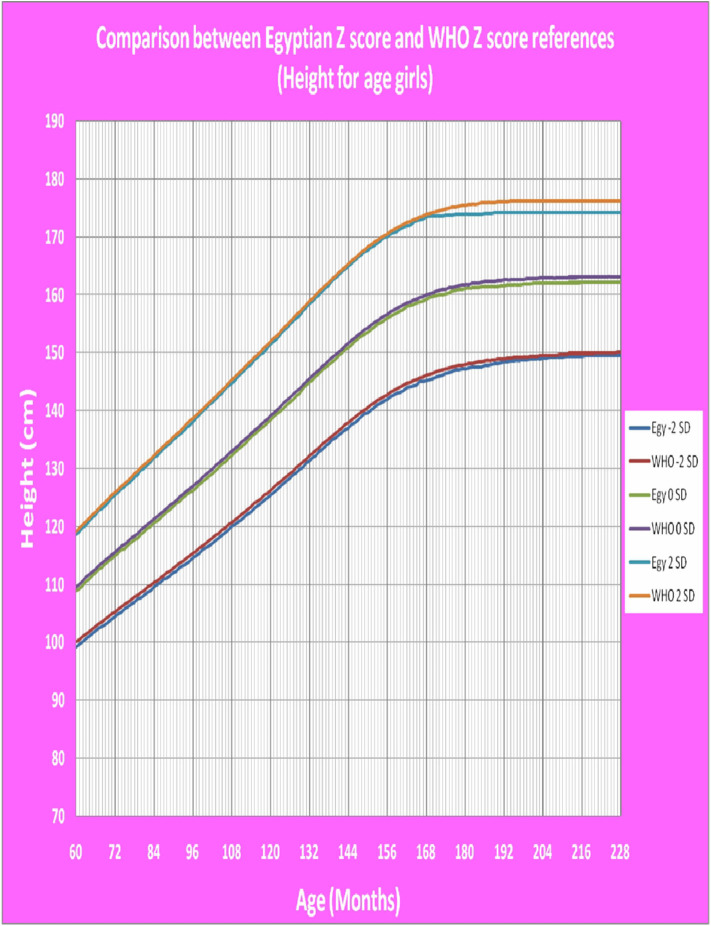
The comparison between Egyptian Z score and WHO Z score references value (Height for age in girls).

Figure 1 shows Egyptian Z score weight for age from 5 to 19 years for boys. Figure 2 shows Egyptian Z score weight for age from 5 to 19 years for girls. Figure 3 shows Egyptian Z score height for age from 5 to 19 years for boys. Figure 4 shows Egyptian Z score height for age from 5 to 19 years for girls. Figure 5 shows the Egyptian Z score BMI for age from 5 year to 19 years for boys. Figure 6 shows the Egyptian Z score BMI for age from 5 year to 19 years for girl. Figure 7 shows the comparison between Egyptian Z score and WHO Z score references value (weight for age in boys). Figure 8 shows the comparison between Egyptian Z score and WHO Z score references value (weight for age in girls). Figure 9 shows the comparison between Egyptian Z score and WHO Z score references value (Height for age in boys). Figure 10 shows the comparison between Egyptian Z score and WHO Z score references value (Height for age in girls).

## Discussion

To assess the growth and nutritional status of a child, percentiles, percent of median, and Z-scores (standard deviation scores) are the available three methods. The Z-score indicates how many standard deviations any value is from the mean (25).

In contrast with a previous research done on 2002, also based on local data, presented the anthropometric parameters using the percentile method (18), our study is the first tool in Egypt to use LMS and Z score growth parameters method that provide more accuracy than percentiles that were used before. When compared with this study in Egypt, the previous study was done only on one governorate of Egypt and was based on percentile, but our report was done in many governorates that were representative to Egypt and based on LMS and Z score method that is more accurate.

Variation in the environmental conditions and the ethnicity factor developed the necessity for the development of tailored charts from representative samples (26, 27), that was and still conducted by many developed (28–30), and developing countries (31–33). In Egypt, this is the first national Z score growth charts for school children and adolescents that was developed in spite of using other charts.

Weight for age was not calculated beyond 10 years of age as during the pubertal growth spurt, the height increases rapidly more than weight making it not an accurate measure of nutritional status beyond 10 years (19).

After comparison of weight, height and BMI for age values of both sexes of the children and adolescents who participated in the present study with the WHO values (19), there was no statistically significant difference between the Egyptian Z score charts and the reference values of WHO. The *P-*values of weight for age in boys and girls were 0.142 and 0.229 (*P* > 0.05), respectively. The *P*-values of height for age in boys and girls were 0.469 and 0.361 (*P* > 0.05), respectively. The *P-*values of BMI for age in boys and girls were 0.492 and 0.316 (*P* > 0.05), respectively. This mean that WHO growth charts may be appropriate for monitoring growth and nutritional status on Egyptian children as the growth pattern in our large population were closer to growth charts of WHO.

WHO Multicenter Growth Reference Study was conducted to provide a single international standard representative for the physiological growth for all children everywhere, regardless of ethnicity, socio-economic status and type of feeding, and our study supports this hypothesis.

## Conclusion

The L, M, and S parameters and Z-scores for Egyptian school children and adolescents presented in this report provide nationally representative reference that will facilitate more accurate assessment of growth and nutritional status of Egyptian children and comparison with other populations under different clinical conditions. In absence of these local charts, we recommend using WHO growth charts.

## Data Availability Statement

All datasets presented in this study are included in the article/ Supplementary Material.

## Ethics Statement

The studies involving human participants were reviewed and approved by Institutional Review Boards (IRB) of the Menoufia Faculty of Medicine. Written informed consent to participate in this study was provided by the participants or their legal guardian/next of kin.

## Author Contributions

AE, ZO, AE-B, and WB: idea and design and data interpretation. AE, ZO, AE-B, MA, AA, AK, HR, GB, AS, WG, and WB: participant enrolment and data collection. AE-B and ZO: manuscript writing. ZK: statistical analysis. AE, ZO, FE-G, DA, MS, AE-B, WB, and ZK: manuscript revision. All authors contributed to the article and approved the submitted version.

## Conflict of Interest

The authors declare that the research was conducted in the absence of any commercial or financial relationships that could be construed as a potential conflict of interest.
