# Supplementary material for: Establishment of Z Score Reference of Growth Parameters for Egyptian School Children and Adolescents Aged From 5 to 19 Years: A Cross Sectional Study
Source: Front Pediatr. 2020 Jul 21;8:368. doi: 10.3389/fped.2020.00368 (PMC7385378; doi:10.3389/fped.2020.00368)
Supplement: Supplementary file 1 [file Data_Sheet_1.PDF]

Supplementary Material

Table (1): shows Egyptian L, M and S parameters and Z score weight for age for boys from 5 years to 10 years.

Weight-for-age BOYS<br>Egyptian Z-score 5 Years to 10 Years											
Y:M	M	Mean	S	L	-3SD	-2SD	-1SD	Median	1SD	2SD	3SD
05:01	61	18.6985	0.13625	-0.1455	12.7	14.5	16.4	18.8	22	25.2	28.9
05:02	62	18.9586	0.13789	-0.1489	12.8	14.6	16.6	19	22.2	25.5	29.1
05:03	63	19.1258	0.13814	-0.1499	12.9	14.7	16.7	19.2	22.4	25.7	29.4
05:04	64	19.3528	0.13951	-0.1502	13.1	14.9	16.9	19.4	22.6	26	29.8
05:05	65	19.5283	0.13989	-0.1555	13.2	15	17.1	19.6	22.8	26.3	30.1
05:06	66	19.7625	0.14008	-0.1589	13.4	15.1	17.3	19.8	23.1	26.6	30.4
05:07	67	19.8222	0.14045	-0.1599	13.5	15.3	17.5	19.9	23.3	26.8	30.8
05:08	68	20.0258	0.14125	-0.1614	13.7	15.4	17.7	20.1	23.5	27.1	31.2
05:09	69	20.2158	0.14201	-0.1635	13.8	15.6	17.8	20.3	23.7	27.4	31.5
05:10	70	20.452	0.14268	-0.1665	14	15.7	18	20.5	23.9	27.7	31.9
05:11	71	20.6586	0.14314	-0.1687	14.1	15.8	18.2	20.7	24.1	28	32.2
06:00	72	20.8562	0.14365	-0.1711	14.2	16	18.3	20.9	24.3	28.3	32.6
06:01	73	21.0369	0.14478	-0.1736	14.3	16.1	18.5	21.1	24.5	28.6	32.9
06:02	74	21.1258	0.14514	-0.1745	14.5	16.2	18.7	21.2	24.7	28.9	33.1
06:03	75	21.3587	0.14695	-0.1799	14.6	16.3	18.9	21.4	24.9	29.2	33.4
06:04	76	21.5247	0.14798	-0.1801	14.7	16.4	19	21.6	25.2	29.5	33.8
06:05	77	21.7952	0.14891	-0.1999	14.8	16.5	19.2	21.8	25.4	29.8	34
06:06	78	22.0368	0.14999	-0.2006	15	16.6	19.4	22	25.6	30.1	34.5
06:07	79	22.102	0.15002	-0.2099	15.1	16.8	19.5	22.2	25.9	30.4	34.8
06:08	80	22.2037	0.15098	-0.2125	15.2	16.9	19.7	22.3	26.1	30.7	35.2
06:09	81	22.4586	0.15125	-0.2265	15.4	17	19.9	22.5	26.4	31	35.8
06:10	82	22.6259	0.15256	-0.2369	15.5	17.2	20	22.7	26.6	31.3	36
06:11	83	22.8745	0.15365	-0.2456	15.6	17.3	20.2	22.9	26.9	31.7	36.4
07:00	84	23.0255	0.15458	-0.2569	15.7	17.4	20.4	23.1	27.1	32	36.8
07:01	85	23.2158	0.15584	-0.2695	15.8	17.6	20.6	23.3	27.4	32.3	37.3
07:02	86	23.3257	0.15695	-0.2789	16	17.7	20.7	23.4	27.7	32.6	37.7
07:03	87	23.5471	0.15789	-0.2896	16.1	17.8	20.9	23.6	27.9	33	38.1
07:04	88	23.7896	0.15874	-0.2987	16.3	18	21	23.8	28.2	33.3	38.6
07:05	89	23.9875	0.15987	-0.3009	16.4	18.1	21.2	24	28.4	33.7	39
07:06	90	24.1258	0.16005	-0.3125	16.5	18.2	21.4	24.2	28.7	34.1	39.5
07:07	91	24.3668	0.16089	-0.3255	16.6	18.4	21.6	24.4	29	34.5	40
07:08	92	24.4565	0.16125	-0.3369	16.8	18.5	21.8	24.5	29.2	34.8	40.5
07:09	93	24.63	0.16258	-0.3456	16.9	18.7	22	24.7	30.1	35.2	41
07:10	94	24.9026	0.16314	-0.3587	17.1	18.8	22.1	24.9	30.3	35.6	41.5
07:11	95	25.0259	0.16458	-0.3625	17.3	18.9	22.3	25.1	30.6	35.9	42
08:00	96	25.2015	0.16589	-0.3789	17.4	19.1	22.5	25.3	30.9	36.3	42.5
08:01	97	25.4632	0.16678	-0.3899	17.5	19.3	22.7	25.5	31.2	36.7	43.1
08:02	98	25.6521	0.16714	-0.3999	17.6	19.5	22.9	25.7	31.5	37.1	43.6
08:03	99	25.8741	0.16825	-0.4128	17.8	19.6	23.1	25.9	31.8	37.5	44.1
08:04	100	26.0215	0.16899	-0.4258	17.9	19.7	23.2	26.1	32	37.9	44.7
08:05	101	26.2015	0.16912	-0.4314	18.1	19.9	23.4	26.3	32.3	38.3	45.2
08:06	102	26.4321	0.16935	-0.4478	18.2	20.1	23.6	26.5	32.5	38.7	45.9
08:07	103	26.6012	0.16978	-0.4547	18.3	20.2	23.8	26.7	32.8	39.1	46.4
08:08	104	26.8745	0.17008	-0.4614	18.5	20.4	24	26.9	33	39.5	47
08:09	105	27.0956	0.17045	-0.4874	18.6	20.6	24.1	27.1	33.3	40	47.6

08:10	106	27.2145	0.17147	-0.4987	18.7	20.8	24.3	27.3	33.6	40.4	48.2
08:11	107	27.4688	0.17199	-0.5265	18.8	20.9	24.5	27.5	33.9	40.8	48.9
09:00	108	27.6653	0.17205	-0.5654	18.9	21	24.7	27.7	34.2	41.3	49.6
09:01	109	27.9028	0.17236	-0.5987	19	21.2	24.9	28	34.5	41.8	50.2
09:02	110	28.1268	0.17258	-0.6147	19.2	21.3	25	28.3	34.8	42.2	50.9
09:03	111	28.4069	0.17301	-0.6357	19.3	21.4	25.1	28.5	35.2	42.7	51.6
09:04	112	28.734	0.17355	-0.6478	19.5	21.6	25.3	28.8	35.5	43.2	52.3
09:05	113	28.8032	0.17366	-0.6531	19.6	21.8	25.5	29	35.9	43.6	53.6
09:06	114	29.125	0.17401	-0.6698	19.7	22	25.7	29.3	36.2	44.1	54.2
09:07	115	29.4563	0.17455	-0.6741	19.9	22.2	25.9	29.6	36.7	44.6	55.1
09:08	116	29.7533	0.17555	-0.6814	20	22.4	26.1	29.9	37	45.1	55.8
09:09	117	30.1255	0.17565	-0.6999	20.2	22.6	26.3	30.2	37.3	45.6	56.5
09:10	118	30.4523	0.17601	-0.7111	20.3	22.8	26.5	30.5	37.6	46.1	57.1
09:11	119	30.8514	0.17655	-0.7222	20.4	23	26.7	30.9	37.9	46.6	57.8
10:00	120	31.1254	0.17788	-0.7325	20.6	23.2	26.9	31.2	38	47.1	58.3

Table (2): shows Egyptian L, M and S parameters and Z score for weight for age for girls from 5 years to 10 years

Weight-for-age GIRLS<br>Egyptian Z-score 5 Years to 10 Years											
Y:M	M	Mean	S	L	-3SD	-2SD	-1SD	Median	1SD	2SD	3SD
05:01	61	18.8563	0.14121	-0.3345	12.4	14.2	16.5	18.9	22.1	26.2	30.3
05:02	62	18.9632	0.14255	-0.3355	12.5	14.3	16.6	19	22.2	26.4	30.6
05:03	63	19.1526	0.14354	-0.3365	12.7	14.4	16.7	19.2	22.4	26.7	30.9
05:04	64	19.3566	0.14456	-0.3379	12.8	14.5	16.8	19.4	22.6	26.9	31.3
05:05	65	19.4586	0.14546	-0.3387	12.9	14.6	17	19.5	22.8	27.1	31.6
05:06	66	19.5035	0.14658	-0.3399	13	14.7	17.1	19.6	23	27.3	31.9
05:07	67	19.7852	0.14789	-0.4002	13.1	14.8	17.3	19.8	23.2	27.5	32.2
05:08	68	19.8563	0.14852	-0.4025	13.2	14.9	17.4	19.9	23.4	27.7	32.5
05:09	69	20.0252	0.14951	-0.4099	13.3	15	17.5	20.1	23.6	27.9	32.9
05:10	70	20.2325	0.15099	-0.4111	13.4	15.2	17.7	20.3	23.9	28.2	33.2
05:11	71	20.4632	0.15148	-0.4125	13.5	15.3	17.9	20.5	24.1	28.5	33.6
06:00	72	20.5963	0.15256	-0.4149	13.6	15.4	18.1	20.7	24.3	28.7	33.9
06:01	73	20.7522	0.15369	-0.4159	13.7	15.5	18.3	20.8	24.4	28.9	34.2
06:02	74	20.9863	0.15478	-0.4201	13.8	15.6	18.4	21	24.6	29.1	34.7
06:03	75	21.1525	0.15528	-0.4255	13.9	15.7	18.5	21.2	24.8	29.3	35
06:04	76	21.2635	0.15654	-0.4265	14	15.8	18.6	21.3	25	29.5	35.4
06:05	77	21.4586	0.15789	-0.4288	14.2	15.9	18.8	21.5	25.3	29.8	35.8
06:06	78	21.6589	0.15825	-0.4299	14.3	16.1	19	21.7	25.4	30	36.1
06:07	79	21.8896	0.15936	-0.4301	14.4	16.2	19.1	21.9	25.7	30.2	36.5
06:08	80	22.0258	0.15999	-0.4322	14.5	16.3	19.3	22.1	26	30.5	37
06:09	81	22.2035	0.16089	-0.4365	14.6	16.5	19.5	22.3	26.3	30.8	37.4
06:10	82	22.3258	0.16101	-0.4375	14.7	16.6	19.6	22.4	26.5	31.1	37.9
06:11	83	22.5688	0.16125	-0.4401	14.8	16.7	19.8	22.6	26.7	31.5	38.2
07:00	84	22.7663	0.16145	-0.4422	14.9	16.9	19.9	22.8	26.9	31.8	38.6
07:01	85	22.8523	0.16189	-0.4435	15	17.1	20	22.9	27	32.1	39
07:02	86	22.9686	0.16205	-0.4455	15.1	17.2	20.1	23	27.2	32.5	39.4
07:03	87	23.2585	0.16266	-0.4499	15.3	17.3	20.3	23.3	27.5	32.8	39.8
07:04	88	23.4585	0.16355	-0.4502	15.4	17.4	20.4	23.5	27.9	33.1	40.3
07:05	89	23.6582	0.16399	-0.4555	15.6	17.5	20.6	23.7	28.2	33.3	41
07:06	90	23.8622	0.16425	-0.4565	15.6	17.6	20.7	23.9	28.5	33.7	41.6
07:07	91	24.0586	0.16899	-0.4575	15.8	17.7	20.8	24.1	28.8	34	42.1
07:08	92	24.2586	0.16925	-0.4581	15.9	17.9	21	24.3	29.1	34.4	42.7
07:09	93	24.4652	0.16951	-0.4599	16	18.1	21.2	24.5	29.4	34.8	43.1
07:10	94	24.5632	0.16978	-0.4602	16.2	18.3	21.4	24.6	29.7	35.2	43.6
07:11	95	24.7556	0.16999	-0.4621	16.4	18.5	21.6	24.8	29.9	35.6	44.1
08:00	96	25.0255	0.17123	-0.4633	16.5	18.7	21.8	25.1	30.2	36	44.7

08:01	97	25.3256	0.17165	-0.4645	16.8	18.9	22	25.4	30.6	36.4	45.3
08:02	98	25.6982	0.17199	-0.4675	17	19	22.2	25.7	30.9	36.8	45.8
08:03	99	25.7896	0.17201	-0.4701	17.2	19.1	22.4	25.9	31.2	39.2	46.2
08:04	100	26.125	0.17225	-0.4745	17.3	19.3	22.7	26.2	31.5	39.5	46.7
08:05	101	26.4525	0.17299	-0.4755	17.5	19.5	23	26.5	31.8	39.9	47.1
08:06	102	26.758	0.17311	-0.4801	17.6	19.7	23.3	26.8	32.1	40.4	47.8
08:07	103	27.0325	0.17335	-0.4825	17.8	19.9	23.5	27.1	32.4	40.9	48.4
08:08	104	27.3662	0.17384	-0.4845	18	20.1	23.7	27.4	32.7	41.1	48.9
08:09	105	27.6225	0.17399	-0.4855	18.1	20.2	23.9	27.7	33	41.8	49.5
08:10	106	27.9533	0.17425	-0.4865	18.2	20.3	24.2	28	33.3	42.4	50
08:11	107	28.3503	0.17445	-0.4912	18.3	20.5	24.4	28.4	33.7	42.9	50.4
09:00	108	28.6333	0.17455	-0.4922	18.5	20.7	24.6	28.7	34	43.3	50.9
09:01	109	28.9563	0.17555	-0.4933	18.6	20.9	24.9	29	34.3	43.8	51.7
09:02	110	29.2325	0.17698	-0.4944	18.7	21.2	25.1	29.3	34.6	44.3	52.4
09:03	111	29.5235	0.17699	-0.4955	18.9	21.4	25.3	29.6	34.9	44.8	53.3
09:04	112	29.868	0.17725	-0.4999	19.1	21.6	25.5	29.9	35.2	45.2	54.1
09:05	113	30.1251	0.17755	-0.5001	19.2	21.8	25.7	30.2	35.4	45.8	54.8
09:06	114	30.4524	0.17765	-0.5025	19.4	22	25.9	30.5	35.8	46.4	56.5
09:07	115	30.7758	0.17775	-0.5075	19.6	22.3	26.2	30.8	36.2	46.9	57.2
09:08	116	31.0258	0.17785	-0.5099	19.8	22.5	26.4	31.1	36.8	47.4	57.9
09:09	117	31.3323	0.17799	-0.5111	19.9	22.7	26.6	31.4	37.2	47.9	58.3
09:10	118	31.6325	0.17802	-0.5125	20	22.9	26.9	31.7	37.7	48.3	59
09:11	119	31.9393	0.17899	-0.5133	20.2	23.1	27.1	32	38.1	48.8	59.6
10:00	120	32.2375	0.17921	-0.5145	20.4	23.4	27.3	32.3	38.4	49.1	60.4

Table (3): shows Egyptian L, M and S parameters and Z score for height for age for boys from 5 years to 19 years

Height-for-age BOYS<br>Egyptian Z-score 5 Years to 19 Years											
Y:M	M	Mean	S	L	-3SD	-2SD	-1SD	Median	1SD	2SD	3SD
05:00	60	109.3	4.88159	1	95.3	100	104.6	109.4	114	118.6	123.5
05:01	61	109.8	5.05613	1	95.8	100.4	105	109.8	114.4	119	123.7
05:02	62	110	4.79432	1	96	100.8	105.5	110.1	115	119.5	124.1
05:03	63	110.6	4.53252	1	96.6	101.2	106	110.7	115.4	120	124.9
05:04	64	111.1	4.58836	1	97	101.6	106.4	111.2	116	120.8	125.5
05:05	65	111.8	4.6442	1	97.4	102.3	107	111.9	116.5	121.3	126
05:06	66	112.1	4.64402	1	98	102.6	107.4	112.2	117.1	122	126.6
05:07	67	112.8	4.89975	1	98.4	103	108	112.9	117.7	122.5	127.2
05:08	68	113.5	5.02762	1	98.8	103.6	108.2	113.6	118.2	123	128
05:09	69	113.9	5.15549	1	99	104	108.9	114	118.8	123.7	128.5
05:10	70	114.4	5.2495	1	99.8	104.3	109.2	114.5	119.5	124.2	129
05:11	71	114.9	5.10849	1	100	105	109.8	115	120	124.8	129.5
06:00	72	115.4	4.96748	1	100.5	105.3	110.2	115.5	120.5	125.4	130.1
06:01	73	115.7	4.96855	1	101	105.6	110.6	115.8	121	126	130.9
06:02	74	116.1	4.96999	1	101.2	106.3	111	116.2	121.5	126.4	131.5
06:03	75	116.7	4.97305	1	101.7	106.6	111.5	116.8	122	127	132
06:04	76	117.2	4.98305	1	102	107	112	117.3	122.6	127.5	132.6
06:05	77	117.8	4.99306	1	102.6	107.5	112.5	117.9	123	128	133
06:06	78	118.3	5.13289	1	103	108	113	118.4	123.5	128.5	133.8
06:07	79	118.7	5.18289	1	103.3	108.3	113.4	118.8	124.1	129	134.4
06:08	80	119	5.20558	1	103.6	108.6	114	119.1	124.5	129.6	134.9
06:09	81	119.6	5.28775	1	104	109	114.3	119.7	125	130.1	135.5
06:10	82	120	5.30669	1	104.2	109.5	114.8	120.1	125.5	130.6	136
06:11	83	120.6	5.32556	1	104.9	110	115.2	120.7	126	131.3	136.5
07:00	84	120.9	5.34945	1	105	110.3	115.6	121	126.6	131.9	137
07:01	85	121.6	5.32445	1	105.6	110.8	116	121.7	127	132.5	137.7
07:02	86	121.9	5.32669	1	105.9	111.3	116.5	122	127.6	133	138.1
07:03	87	122.5	5.31189	1	106.2	111.6	117	122.6	128	133.4	138.8
07:04	88	122.9	5.32669	1	106.4	112	117.4	123	128.6	134	139.3
07:05	89	123.4	5.3807	1	107	112.4	117.8	123.5	129	134.5	140
07:06	90	123.9	5.4107	1	107.2	112.9	118.3	124	129.5	135	140.3
07:07	91	124.4	5.45898	1	107.6	113.3	118.6	124.5	130	135.5	141
07:08	92	124.9	5.50749	1	108	113.6	119	125	130.6	136	141.5
07:09	93	125.2	5.60748	1	108.6	114	119.5	125.3	131	136.6	142
07:10	94	125.7	5.70555	1	108.9	114.3	120	125.8	131.4	137	142.5
07:11	95	126	5.80693	1	109.2	114.7	120.3	126.1	132	137.6	143.1
08:00	96	126.7	5.86112	1	109.6	115.2	120.8	126.8	132.5	138	143.6
08:01	97	126.9	5.63987	1	110	115.7	121.2	127	133	138.5	144.2

08:02	98	127.4	5.25846	1	110.3	115.9	121.6	127.5	133.4	139	144.9
08:03	99	127.9	5.78527	1	110.6	116.2	122	128	133.9	139.5	145.3
08:04	100	128.2	5.79365	1	111	116.5	122.5	128.3	134.3	140	146
08:05	101	128.9	5.80245	1	111.3	117	123	129	134.8	140.6	146.5
08:06	102	129.2	5.81614	1	111.6	117.5	123.4	129.3	135.3	141	147
08:07	103	129.8	5.85963	1	112	118	123.6	129.9	135.6	141.5	147.5
08:08	104	130	5.88471	1	112.3	118.3	124	130.1	136.2	142	148
08:09	105	130.6	5.89586	1	112.7	118.6	124.5	130.7	136.7	142.7	148.5
08:10	106	130.9	5.91258	1	113	119	125	131	137	143	149
08:11	107	131.4	5.93654	1	113.4	119.3	125.4	131.5	137.5	143.5	149.5
09:00	108	131.9	5.94979	1	113.7	119.6	125.9	132	138	144.2	150
09:01	109	132.4	5.20369	1	114	120	126.2	132.5	138.5	144.7	150.5
09:02	110	132.7	5.30254	1	114.3	120.5	126.4	132.8	139	145	151.1
09:03	111	133.1	6.32289	1	114.9	121	127	133.2	139.4	145.5	151.5
09:04	112	133.7	6.32289	1	115	121.3	127.4	133.8	139.8	146	152.2
09:05	113	133.9	6.34874	1	115.6	121.6	127.8	134	140.2	146.5	152.5
09:06	114	134.5	6.35916	1	115.8	122	128.2	134.6	140.9	147	153.2
09:07	115	134.9	6.47681	1	116	122.4	128.5	135	141.2	147.6	153.9
09:08	116	135.4	6.45874	1	116.6	122.7	129	135.5	141.8	148	154.2
09:09	117	135.9	6.46296	1	116.8	123	129.3	136	142.4	148.6	154.9
09:10	118	136.1	6.24934	1	117.2	123.5	129.9	136.2	142.8	149	155.5
09:11	119	136.4	6.25698	1	117.5	124	130.2	136.5	143	149.5	156
10:00	120	137	6.28541	1	118	124.3	130.6	137.1	143.2	150.1	156.5
10:01	121	137.4	6.29665	1	118.3	124.7	131	137.5	144	150.6	157
10:02	122	137.9	6.40236	1	118.6	125	131.5	138	144.6	151	157.5
10:03	123	138.5	6.42356	1	119	125.5	131.7	138.6	145	151.5	158
10:04	124	138.9	6.45255	1	119.3	125.8	132.3	139	145.5	152	158.5
10:05	125	139.4	6.25845	1	119.7	126	132.6	139.5	146	152.5	159
10:06	126	139.7	6.45901	1	120	126.6	133	139.8	146.2	153	159.6
10:07	127	140	6.46987	1	120.4	127	133.5	140.1	146.9	153.5	160
10:08	128	140.7	6.49654	1	120.7	127.4	134	140.8	147.2	154	160.7
10:09	129	140.9	6.50756	1	121	127.8	134.3	141	147.9	154.5	161.3
10:10	130	141.4	6.78526	1	121.3	128	134.7	141.5	148.3	155.1	161.8
10:11	131	141.9	6.2584	1	121.8	128.3	135	142	148.9	155.5	162.5
11:00	132	142.4	6.93484	1	122	129	135.6	142.5	149.3	156.2	162.9
11:01	133	142.9	6.82548	1	122.4	129.3	136	143	149.6	156.7	163.5
11:02	134	143.4	6.32548	1	123	129.7	136.6	143.5	150.3	157.2	164
11:03	135	143.9	6.70349	1	123.4	130	137	144	150.8	157.8	164.5
11:04	136	144.3	6.85239	1	123.7	130.6	137.5	144.4	151.2	158.1	165
11:05	137	144.9	6.99658	1	124	131	137.9	145	151.8	158.9	165.5
11:06	138	145.4	7.06473	1	124.6	131.5	138.3	145.5	152.4	159.4	166.1
11:07	139	145.9	7.06584	1	125	131.9	138.8	146	152.9	160	166.9
11:08	140	146.3	7.07223	1	125.3	132.2	139.2	146.4	153.5	160.4	167.5
11:09	141	146.9	7.11258	1	125.7	132.6	139.6	147	154	161	168
11:10	142	147.4	7.11256	1	126	133.2	140.3	147.5	154.5	161.6	168.5
11:11	143	147.9	7.1528	1	126.7	133.6	140.7	148	155	162.2	169.1

12:00	144	148.3	7.11197	1	127	134	141.3	148.4	155.7	162.9	169.8
12:01	145	148.9	7.12369	1	127.5	134.5	141.6	149	156	163.4	170.5
12:02	146	149.4	7.13548	1	128	135	142.2	149.5	156.7	164	171
12:03	147	149.9	7.19038	1	128.6	135.5	142.8	150	157.2	164.5	171.6
12:04	148	150.6	7.17258	1	129	136	143.2	150.7	158	165.1	172.6
12:05	149	151.1	7.18936	1	129.5	136.6	143.7	151.2	158.5	165.9	173
12:06	150	151.7	7.15953	1	130	137	144.5	151.8	159	166.5	173.8
12:07	151	152.4	7.25896	1	130.5	137.6	145	152.5	159.7	167	174.5
12:08	152	152.9	7.24852	1	131	138.2	145.5	153	160.2	167.7	175
12:09	153	153.4	7.48601	1	131.7	138.7	146	153.5	161	168.5	175.8
12:10	154	154	7.25895	1	132	139.3	146.8	154.1	161.5	169	176.5
12:11	155	154.8	7.4191	1	132.5	139.9	147.2	154.9	162.1	169.8	177
13:00	156	155.3	7.4391	1	133	140.3	147.7	155.4	163	170.2	177.8
13:01	157	155.9	7.49369	1	133.4	141	148.4	156	163.5	171	178.5
13:02	158	156.6	7.51582	1	134	141.5	149	156.7	164.2	171.8	179.1
13:03	159	157.1	7.55768	1	134.5	142	149.5	157.2	164.9	172.5	180
13:04	160	157.9	7.56936	1	135	142.6	150.2	158	165.5	173	180.5
13:05	161	158.3	7.57237	1	135.6	143.2	150.7	158.4	166	173.8	181.3
13:06	162	158.9	7.58317	1	136.2	143.6	151.4	159	166.7	174.4	182
13:07	163	159.6	7.54852	1	136.7	144.2	152	159.7	167.2	175	182.5
13:08	164	160.1	7.56958	1	137.2	145	152.5	160.2	168	175.5	183.3
13:09	165	160.9	7.5476	1	137.9	145.3	153	161	168.5	176.2	184
13:10	166	161.4	7.61258	1	138.3	146	153.6	161.5	169	177	184.6
13:11	167	161.9	7.64365	1	138.9	146.5	154	162	169.7	177.5	185
14:00	168	162.4	7.62587	1	139.3	147	154.7	162.5	170.2	178	185.9
14:01	169	162.9	7.69325	1	139.7	147.6	155.3	163	170.8	178.5	186.5
14:02	170	163.4	7.2646	1	140.2	148	155.6	163.5	171.5	179.1	187
14:03	171	164.1	7.51323	1	140.8	148.5	156.2	164.2	172	179.7	187.5
14:04	172	164.4	7.65285	1	141.3	149	156.8	164.5	172.5	180.3	188
14:05	173	165	7.70958	1	141.7	149.6	157.3	165.1	173	180.8	188.5
14:06	174	165.4	7.74984	1	142.3	150	157.7	165.5	173.7	181.3	189
14:07	175	166	7.65985	1	142.7	150.4	158.2	166.1	174	181.9	189.5
14:08	176	166.4	7.58325	1	143	151	158.6	166.5	174.5	182.5	190
14:09	177	166.9	7.60563	1	143.6	151.2	159	167	175.1	182.9	190.5
14:10	178	167.3	7.61258	1	144	151.6	159.6	167.4	175.5	183.1	191
14:11	179	167.9	7.63258	1	144.3	152	160	168	175.9	183.7	191.5
15:00	180	168.4	7.64829	1	144.7	152.6	160.5	168.5	176.4	184	192
15:01	181	168.7	7.62585	1	145	153	160.7	168.8	176.8	184.5	192.3
15:02	182	168.9	7.69584	1	145.5	153.3	161	169	177.1	185	192.8
15:03	183	169.4	7.68082	1	146	153.7	161.5	169.5	177.4	185.2	193
15:04	184	169.9	7.68082	1	146.3	154	162	170	177.9	185.5	193.5
15:05	185	170	7.6582	1	146.6	154.5	162.3	170.1	178.1	186	193.8
15:06	186	170.4	7.69607	1	147	154.8	162.6	170.5	178.4	186.2	194
15:07	187	170.9	7.70542	1	147.4	155	163	171	178.9	186.7	194.3
15:08	188	171.1	7.74126	1	147.7	155.4	163.2	171.2	179.2	187	194.8
15:09	189	171.4	7.74219	1	148	155.7	163.5	171.5	179.5	187.3	195

15:10	190	171.6	7.63259	1	148.3	156	163.7	171.7	179.7	187.5	195.2
15:11	191	171.9	7.65294	1	148.6	156.2	164	172	180	187.8	195.5
16:00	192	172.1	7.51079	1	148.9	156.6	164.2	172.2	180.2	188	195.7
16:01	193	172.3	7.88363	1	149	156.8	164.6	172.4	180.4	188.2	195.7
16:02	194	172.6	7.99258	1	149.4	157	164.8	172.7	180.6	188.5	195.7
16:03	195	172.9	8.18876	1	149.6	157.3	165	173	181	188.7	195.7
16:04	196	173.1	9.02584	1	149.9	157.6	165.2	173.2	181.1	188.9	195.7
16:05	197	173.3	7.44582	1	150	157.8	165.5	173.4	181.3	189	195.7
16:06	198	173.5	7.77163	1	150.3	158	165.7	173.6	181.4	189.1	195.7
16:07	199	173.7	7.25841	1	150.6	158.2	166	173.8	181.6	189.2	195.7
16:08	200	173.9	7.36952	1	150.8	158.4	166.2	174	181.9	189.5	195.7
16:09	201	174	7.73217	1	151	158.6	166.4	174.1	182	189.6	195.7
16:10	202	174.2	7.65285	1	151.2	158.8	166.5	174.3	182.1	189.7	195.7
16:11	203	174.3	7.60258	1	151.4	159	166.6	174.4	182.2	189.8	195.7
17:00	204	174.6	7.63469	1	151.6	159.1	166.7	174.7	182.4	189.9	195.7
17:01	205	174.7	7.47528	1	151.8	159.2	166.9	174.8	182.5	190	195.7
17:02	206	174.8	7.41583	1	151.9	159.3	167	174.9	182.6	190	195.7
17:03	207	174.9	7.51005	1	152	159.4	167.1	175	182.7	190.2	195.7
17:04	208	175	8.01253	1	152.1	159.6	167.2	175.1	182.7	190.2	195.7
17:05	209	175.1	8.12582	1	152.2	159.7	167.3	175.2	182.8	190.2	195.7
17:06	210	175.2	8.21785	1	152.3	159.8	167.4	175.3	182.8	190.3	195.7
17:07	211	175.2	7.56932	1	152.4	160	167.5	175.3	182.9	190.3	195.7
17:08	212	175.3	7.25899	1	152.5	160.1	167.6	175.4	183	190.4	195.7
17:09	213	175.3	7.36147	1	152.6	160.2	167.7	175.4	183	190.4	195.7
17:10	214	175.3	7.25814	1	152.6	160.3	167.8	175.4	183	190.5	195.7
17:11	215	175.4	7.26149	1	152.7	160.3	167.9	175.5	183.1	190.5	195.7
18:00	216	175.4	7.31851	1	152.8	160.4	168	175.5	183.1	190.5	195.7
18:01	217	175.5	8.12584	1	152.9	160.5	168.1	175.6	183.1	190.5	195.7
18:02	218	175.6	8.13693	1	152.9	160.6	168.2	175.7	183.2	190.5	195.7
18:03	219	175.6	8.29768	1	153	160.6	168.2	175.7	183.2	190.5	195.7
18:04	220	175.7	8.12548	1	153.1	160.7	168.3	175.8	183.2	190.5	195.7
18:05	212	175.7	8.36254	1	153.2	160.8	168.3	175.8	183.3	190.6	195.7
18:06	222	175.7	7.19216	1	153.3	160.8	168.3	175.8	183.3	190.6	195.7
18:07	223	175.8	7.12584	1	153.4	160.8	168.3	175.8	183.3	190.6	195.7
18:08	224	175.8	7.36258	1	153.5	160.9	168.4	175.9	183.3	190.7	195.7
18:09	225	175.8	7.25757	1	153.6	160.9	168.4	175.9	183.3	190.7	195.7
18:10	226	175.8	7.23695	1	153.8	161	168.5	175.9	183.3	190.7	195.7
18:11	227	175.8	7.23584	1	153.8	161	168.5	175.9	183.3	190.7	195.7
19:00	228	175.8	7.24369	1	153.8	161	168.5	175.9	183.3	190.7	195.7

Table (4): shows Egyptian L, M and S parameters and Z score for height for age for girls from 5 years to 19 years

Height-for-age GIRLS Egyptian Z-score 5Years to 19 Years											
Y:M	M	Mean	S	L	-3SD	-2SD	-1SD	Median	1SD	2SD	3SD
05:01	61	109	5.01271	1	94.4	99.2	104.1	109	114	118.7	123.5
05:02	62	109.2	5.01258	1	95	99.7	104.5	109.3	114.3	119.1	124
05:03	63	109.8	5.01281	1	95.3	100.2	105	109.9	115	119.9	124.8
05:04	64	110.5	5.32658	1	95.6	100.6	105.5	110.5	115.5	120.2	125.3
05:05	65	111	5.32587	1	96.2	101	106	111	116	121	126
05:06	66	111.5	5.33867	1	96.6	101.5	106.3	111.5	116.7	121.6	126.5
05:07	67	111.9	5.02587	1	97	102	107	112	117	122	127
05:08	68	112.5	5.02658	1	97.4	102.4	107.3	112.6	117.8	122.8	127.6
05:09	69	112.9	5.02021	1	97.7	102.8	107.9	113	118.2	123.1	128.2
05:10	70	113.5	5.32147	1	98.1	103.1	108.2	113.6	118.8	123.6	128.9
05:11	71	113.9	5.25874	1	98.6	103.6	108.9	114	119.1	124.2	129.4
06:00	72	114.4	5.31711	1	99	104	109.1	114.5	119.8	125	130
06:01	73	115	5.26584	1	99.3	104.5	109.7	115	120.4	125.5	130.7
06:02	74	115.5	5.21478	1	99.6	105	110	115.5	120.8	126	131
06:03	75	115.9	5.08827	1	100	105.3	110.4	116	121.2	126.5	131.7
06:04	76	116.1	5.02587	1	100.5	105.9	111	116.2	121.9	127	132.5
06:05	77	116.7	5.14847	1	101	106.2	111.5	116.8	122.3	127.5	132.9
06:06	78	117.2	5.45681	1	101.2	106.6	112	117.3	122.8	128	133.5
06:07	79	117.6	5.22654	1	101.6	107	112.3	117.7	123.2	128.5	134
06:08	80	118	5.22963	1	102	107.5	112.9	118	123.6	129	134.5
06:09	81	118.5	5.22416	1	102.4	107.7	113.2	118.6	124.2	129.8	135
06:10	82	119	5.22874	1	102.8	108.1	113.7	119.1	124.8	130.1	135.5
06:11	83	119.6	5.22985	1	103.1	108.7	114	119.7	125.4	130.7	136.1
07:00	84	120	5.39606	1	103.6	109	114.5	120	125.9	131.2	136.8
07:01	85	120.6	5.39654	1	104	109.5	115	120.7	126.2	131.9	137.4
07:02	86	121	5.60258	1	104.3	110	115.4	121	126.8	132.3	137.9
07:03	87	121.4	5.60727	1	104.7	110.3	116	121.5	127.4	132.9	138.5
07:04	88	122	5.45789	1	105.2	110.9	116.3	122	127.9	133.5	139
07:05	89	122.4	5.25632	1	105.6	111.1	116.7	122.5	128.3	134	139.5
07:06	90	122.9	5.76285	1	106	111.6	117.2	123	128.9	134.4	140
07:07	91	123.4	5.21487	1	106.5	112	117.7	123.5	129.5	135	140.7
07:08	92	124	5.28514	1	106.8	112.3	118	124	129.9	135.5	141.1
07:09	93	124.3	5.56728	1	107.1	113	118.5	124.4	130.4	136	141.7
07:10	94	124.9	5.24854	1	107.6	113.3	119	125	130.7	136.5	142.3
07:11	95	125.4	5.65987	1	108	113.6	119.5	125.4	131.3	137	143
08:00	96	125.9	5.7613	1	108.3	114.1	120	126	132	137.5	143.3
08:01	97	126.1	6.21525	1	108.7	114.6	120.5	126.2	132.5	138.1	144
08:02	98	126.6	6.23584	1	109.1	115	121	126.7	133	138.7	144.5

08:03	99	127.2	6.21232	1	109.6	115.5	121.3	127.3	133.4	139.3	145.2
08:04	100	127.7	6.06325	1	110	116	121.8	127.8	134	139.9	145.8
08:05	101	128.2	6.02582	1	110.4	116.3	122.2	128.3	134.3	140.4	146.3
08:06	102	128.6	6.06359	1	110.8	116.7	122.6	128.7	135	141	147
08:07	103	129.1	6.35924	1	111.2	117.1	123.3	129.2	135.6	141.5	147.5
08:08	104	129.6	6.32584	1	111.7	117.6	123.8	129.7	136	142	148
08:09	105	130.2	6.38924	1	112	118	124.2	130.3	136.6	142.6	148.7
08:10	106	130.7	6.02584	1	112.4	118.5	124.6	130.8	137	143	149.1
08:11	107	131.1	6.02359	1	113	119	125	131.2	137.6	143.8	149.8
09:00	108	131.7	6.03576	1	113.3	119.5	125.6	131.8	138	144.3	150.3
09:01	109	132.1	6.32587	1	113.8	120	126	132.2	138.7	144.7	151
09:02	110	132.7	6.25478	1	114.2	120.4	126.5	132.8	139.1	145.2	151.5
09:03	111	133.2	6.29722	1	114.6	120.9	127	133.3	139.7	146	152
09:04	112	133.7	6.25584	1	115	121.2	127.4	133.8	140.3	146.5	152.5
09:05	113	134.1	6.32547	1	115.5	121.7	128	134.2	140.9	147	153.2
09:06	114	134.8	6.86152	1	116	122.2	128.5	134.9	141.3	147.7	153.9
09:07	115	135.4	6.25874	1	116.5	122.6	129	135.5	141.8	148	154.4
09:08	116	136	6.36985	1	117	123.2	129.5	136	142.4	148.8	155
09:09	117	136.4	6.64078	1	117.3	123.6	130	136.5	143	149.2	155.7
09:10	118	137	6.32145	1	117.7	124	130.5	137	143.5	149.9	156.1
09:11	119	137.2	6.32652	1	118.1	124.6	131	137.3	144	150.2	156.7
10:00	120	137.9	6.26325	1	118.6	125	131.5	138	144.5	151	157.3
10:01	121	138.4	6.23568	1	119	125.4	132	138.5	145	151.5	158
10:02	122	139	6.25874	1	119.5	126	132.5	139	145.5	152	158.6
10:03	123	139.4	6.76651	1	120	126.4	133	139.5	146.2	152.5	159
10:04	124	139.9	6.25142	1	120.5	127	133.4	140	146.7	153.2	159.8
10:05	125	140.4	6.25143	1	121	127.4	134	140.5	147.2	153.9	160.4
10:06	126	140.9	6.94455	1	121.3	128	134.4	141	147.7	154.4	161
10:07	127	141.4	6.32145	1	122	128.4	135	141.5	148.5	155	161.5
10:08	128	142	6.31542	1	122.3	129	135.5	142	149	155.5	162
10:09	129	142.5	6.82988	1	122.8	129.4	136	142.6	149.5	156	162.5
10:10	130	143	7.25874	1	123.2	130	136.5	143	150	156.5	163.2
10:11	131	143.7	7.25147	1	123.7	130.3	137	143.8	150.5	157.2	163.9
11:00	132	144.3	7.2237	1	124.3	131	137.4	144.4	151	157.9	164.5
11:01	133	144.8	7.22584	1	124.7	131.3	138	144.9	151.6	158.5	165
11:02	134	145.2	7.33698	1	125.1	132	138.6	145.3	152.1	159	165.5
11:03	135	146	6.79107	1	125.7	132.3	139	146	152.8	159.5	166.2
11:04	136	146.5	7.04235	1	126.1	133	139.6	146.5	153.4	160	166.8
11:05	137	147	7.04524	1	126.5	133.3	140	147	154	160.6	167.5
11:06	138	147.4	7.04072	1	127	134	140.6	147.5	154.4	161.2	168
11:07	139	147.9	7.02587	1	127.5	134.4	141	148	155	161.8	168.5
11:08	140	148.3	7.36985	1	128	135	141.6	148.4	155.4	162.5	169
11:09	141	148.9	7.3796	1	128.5	135.3	142	149	156	162.9	169.5
11:10	142	149.5	7.25874	1	129	135.8	142.7	149.5	156.7	163.5	170.1
11:11	143	149.9	7.32654	1	129.4	136.3	143	150	157	164	170.7
12:00	144	150.5	6.91346	1	130	136.8	143.5	150.6	157.5	164.5	171.3

12:01	145	150.9	6.99874	1	130.3	137.1	144	151	158	165	171.8
12:02	146	151.3	6.22584	1	130.8	137.7	144.5	151.4	158.5	165.4	172.3
12:03	147	151.9	6.99624	1	131.1	138	145	152	159	166	172.9
12:04	148	152.4	6.25147	1	131.6	138.5	145.4	152.5	159.5	166.5	173.2
12:05	149	153	6.25148	1	132	139	146	153	160	167	173.6
12:06	150	153.2	7.69385	1	132.5	139.5	146.3	153.3	160.5	167.5	174.2
12:07	151	153.5	7.06584	1	133	139.7	146.8	153.6	160.8	167.7	174.8
12:08	152	154	7.25147	1	133.3	140.2	147	154	161.3	168.2	175
12:09	153	154.4	7.06555	1	133.6	140.6	147.5	154.5	161.8	168.5	175.5
12:10	154	155	7.04258	1	134	141	148	155	162	169	176
12:11	155	155.1	7.04528	1	134.3	141.3	148.4	155.2	162.5	169.4	176.3
13:00	156	155.5	7.04015	1	134.7	141.6	148.7	155.6	162.9	169.9	176.7
13:01	157	156	6.32145	1	135	142	149	156	163.1	170	177
13:02	158	156.3	6.93258	1	135.4	142.4	149.3	156.4	163.5	170.5	177.5
13:03	159	156.6	6.93707	1	135.7	142.7	149.6	156.7	163.9	170.8	177.8
13:04	160	156.9	7.02548	1	136	143	150	157	164.2	171	177.9
13:05	161	157.1	7.02584	1	136.4	143.2	150.3	157.2	164.5	171.5	178
13:06	162	157.4	7.05622	1	136.6	143.6	150.6	157.5	164.8	171.8	178.1
13:07	163	157.9	7.02587	1	137	144	150.9	158	165	172	178.1
13:08	164	158.1	7.02591	1	137.1	144.1	151	158.2	165.4	172.1	178.1
13:09	165	158.3	7.21633	1	137.3	144.3	151.2	158.4	165.6	172.5	178.1
13:10	166	158.5	6.96325	1	137.4	144.5	151.6	158.6	165.9	172.8	178.1
13:11	167	158.7	6.39587	1	137.8	145	151.8	158.8	166	173	178.2
14:00	168	158.9	6.9325	1	138.1	145.1	152	159	166.1	173.1	178.2
14:01	169	159.1	6.66547	1	138.3	145.2	152.2	159.2	166.2	173.5	178.3
14:02	170	159.4	6.56441	1	138.5	145.4	152.5	159.5	166.5	173.6	178.3
14:03	171	159.6	6.78079	1	138.7	145.6	152.7	159.7	166.8	173.6	178.4
14:04	172	159.8	6.45871	1	139	145.8	152.8	159.9	167	173.7	178.4
14:05	173	159.9	6.25147	1	139.3	146	153	160	167.2	173.7	178.5
14:06	174	160	6.72809	1	139.4	146.2	153.2	160.1	167.3	173.7	178.5
14:07	175	160.1	6.25147	1	139.6	146.4	153.4	160.2	167.5	173.8	178.6
14:08	176	160.3	6.25874	1	139.7	146.6	153.6	160.4	167.7	173.8	178.6
14:09	177	160.5	6.6758	1	139.8	146.8	153.7	160.6	167.8	173.8	178.7
14:10	178	160.7	6.25145	1	140	147	153.8	160.8	167.9	173.9	178.7
14:11	179	160.8	6.25147	1	140.1	147.1	153.9	160.9	168	173.9	178.8
15:00	180	160.9	6.82588	1	140.2	147.2	154	161	168	173.9	178.8
15:01	181	161	6.25847	1	140.3	147.3	154.1	161.1	168.1	174	178.9
15:02	182	161.1	6.25841	1	140.4	147.4	154.2	161.2	168.2	174	178.9
15:03	183	161.2	6.63765	1	140.5	147.5	154.3	161.2	168.3	174	179
15:04	184	161.2	6.25847	1	140.7	147.5	154.4	161.3	168.4	174	179.1
15:05	185	161.2	6.23154	1	140.7	147.5	154.5	161.3	168.5	174	179.1
15:06	186	161.3	6.64982	1	141	147.6	154.6	161.4	168.5	174	179.2
15:07	187	161.3	6.62587	1	141.1	147.8	154.6	161.4	168.6	174.1	179.2
15:08	188	161.3	6.62852	1	141.2	147.9	154.7	161.4	168.6	174.1	179.2
15:09	189	161.4	6.62815	1	141.3	148	154.7	161.5	168.6	174.1	179.3
15:10	190	161.4	6.52698	1	141.4	148.1	154.8	161.5	168.7	174.2	179.3

15:11	191	161.4	6.52369	1	141.5	148.2	154.8	161.5	168.7	174.2	179.4
16:00	192	161.5	6.5274	1	141.6	148.3	154.8	161.6	168.7	174.2	179.4
16:01	193	161.5	6.41789	1	141.7	148.4	155	161.6	168.8	174.2	179.5
16:02	194	161.6	6.41654	1	141.8	148.5	155	161.7	168.8	174.2	179.5
16:03	195	161.6	6.41905	1	141.9	148.6	155	161.7	168.8	174.2	179.6
16:04	196	161.7	6.42654	1	142	148.6	155.1	161.8	168.8	174.2	179.6
16:05	197	161.7	6.42587	1	142.1	148.7	155.1	161.8	168.8	174.2	179.7
16:06	198	161.7	6.42287	1	142.2	148.7	155.2	161.8	168.9	174.2	179.7
16:07	199	161.8	6.50258	1	142.3	148.8	155.2	161.9	168.9	174.2	179.8
16:08	200	161.8	6.32548	1	142.4	148.8	155.2	161.9	168.9	174.2	179.8
16:09	201	161.8	6.50674	1	142.4	148.9	155.3	161.9	168.9	174.2	179.9
16:10	202	162	6.39584	1	142.5	148.9	155.3	162	168.9	174.2	179.9
16:11	203	162	6.39654	1	142.5	149	155.3	162	168.9	174.2	180
17:00	204	162	6.39211	1	142.5	149	155.4	162	168.9	174.2	180
17:01	205	162	6.47456	1	142.6	149.1	155.4	162.1	169	174.2	180
17:02	206	162	6.47582	1	142.6	149.1	155.4	162.1	169	174.2	180
17:03	207	162	6.47963	1	142.6	149.2	155.4	162.1	169	174.2	180
17:04	208	162	6.17546	1	142.6	149.2	155.5	162.1	169	174.2	180
17:05	209	162	6.17951	1	142.6	149.3	155.5	162.1	169.1	174.2	180
17:06	210	162	6.17637	1	142.6	149.3	155.5	162.1	169.1	174.2	180
17:07	211	162	6.16524	1	142.7	149.3	155.6	162.1	169.1	174.2	180
17:08	212	162	6.16258	1	142.7	149.4	155.6	162.1	169.1	174.2	180
17:09	213	162	6.16214	1	142.7	149.4	155.6	162.1	169.1	174.2	180
17:10	214	162	6.16258	1	142.7	149.4	155.6	162.1	169.1	174.2	180
17:11	215	162	6.15978	1	142.7	149.4	155.6	162.1	169.1	174.2	180
18:00	216	162	6.15977	1	142.7	149.4	155.7	162.1	169.1	174.2	180
18:01	217	162.1	6.18528	1	142.7	149.4	155.7	162.2	169.1	174.2	180
18:02	218	162.1	6.18254	1	142.7	149.5	155.7	162.2	169.1	174.2	180
18:03	219	162.1	6.18673	1	142.8	149.5	155.7	162.2	169.1	174.2	180
18:04	220	162.1	6.19321	1	142.8	149.5	155.7	162.2	169.1	174.2	180
18:05	221	162.1	6.19632	1	142.8	149.5	155.8	162.2	169.2	174.2	180
18:06	222	162.1	6.19187	1	142.8	149.5	155.8	162.2	169.2	174.2	180
18:07	223	162.1	6.19187	1	142.8	149.5	155.8	162.2	169.2	174.2	180
18:08	224	162.1	6.19487	1	142.8	149.5	155.8	162.2	169.2	174.2	180
18:09	225	162.1	6.19187	1	142.8	149.5	155.8	162.2	169.2	174.2	180
18:10	226	162.1	6.19187	1	142.8	149.5	155.8	162.2	169.2	174.2	180
18:11	227	162.1	6.47949	1	142.8	149.5	155.8	162.2	169.2	174.2	180
19:00	228	162.1	6.47949	1	142.8	149.5	155.8	162.2	169.2	174.2	180

Table (5): shows Egyptian L, M and S parameters and Z score for BMI for age for boys from 5 years to 19 years

BMI-for-age BOYS<br>Egyptian Z-score 5Years to 19 Years											
Y:M	M	Mean	S	L	-3SD	-2SD	-1SD	Median	1SD	2SD	3SD
05:01	61	15.7062	0.08502	-0.3611	12.5	13.3	14.5	15.7	17.1	19.8	21.3
05:02	62	15.7359	0.08525	-0.3633	12.5	13.3	14.5	15.7	17.2	19.8	21.3
05:03	63	15.7456	0.08539	-0.3655	12.5	13.3	14.5	15.7	17.2	19.8	21.4
05:04	64	15.7586	0.08555	-0.3699	12.5	13.3	14.5	15.7	17.2	19.8	21.4
05:05	65	15.7685	0.08599	-0.3744	12.5	13.3	14.5	15.7	17.2	19.8	21.5
05:06	66	15.7758	0.08602	-0.3777	12.5	13.3	14.5	15.7	17.2	19.9	21.5
05:07	67	15.7862	0.08609	-0.3788	12.5	13.3	14.5	15.7	17.2	19.9	21.6
05:08	68	15.7895	0.08623	-0.3799	12.5	13.3	14.5	15.7	17.2	19.9	21.6
05:09	69	15.7935	0.08665	-0.3801	12.5	13.3	14.5	15.7	17.2	19.9	21.6
05:10	70	15.8052	0.08688	-0.3822	12.5	13.3	14.5	15.8	17.2	19.9	21.7
05:11	71	15.8125	0.08711	-0.3844	12.5	13.3	14.5	15.8	17.2	20	21.7
06:00	72	15.8687	0.08755	-0.3855	12.5	13.3	14.5	15.8	17.3	20	21.7
06:01	73	15.8789	0.08745	-0.3865	12.5	13.3	14.5	15.8	17.3	20	21.7
06:02	74	15.8896	0.08765	-0.3875	12.6	13.3	14.5	15.8	17.3	20	21.8
06:03	75	15.8938	0.08799	-0.3885	12.6	13.3	14.5	15.8	17.3	20.1	21.8
06:04	76	15.9085	0.08803	-0.3895	12.6	13.3	14.5	15.9	17.3	20.1	21.9
06:05	77	15.9093	0.08833	-0.3899	12.6	13.3	14.5	15.9	17.3	20.1	21.9
06:06	78	15.9185	0.08845	-0.3901	12.6	13.3	14.6	15.9	17.4	20.2	21.9
06:07	79	15.9356	0.08861	-0.3922	12.6	13.3	14.6	15.9	17.4	20.2	22
06:08	80	15.9756	0.08877	-0.3935	12.6	13.3	14.6	15.9	17.4	20.2	22.1
06:09	81	15.9985	0.08888	-0.3945	12.6	13.3	14.6	15.9	17.4	20.3	22.2
06:10	82	16.0159	0.08901	-0.3956	12.6	13.3	14.6	16	17.4	20.3	22.3
06:11	83	16.0358	0.08945	-0.3966	12.6	13.3	14.6	16	17.4	20.4	22.4
07:00	84	16.0458	0.08955	-0.3978	12.7	13.3	14.6	16	17.5	20.4	22.5
07:01	85	16.0689	0.08999	-0.3999	12.7	13.3	14.6	16	17.6	20.5	22.6
07:02	86	16.0786	0.09001	-0.4009	12.7	13.3	14.7	16	17.6	20.5	22.7
07:03	87	16.1158	0.09039	-0.4022	12.7	13.3	14.7	16.1	17.7	20.6	22.8
07:04	88	16.135	0.09111	-0.4125	12.7	13.4	14.7	16.1	17.7	20.7	22.9
07:05	89	16.1456	0.09123	-0.4258	12.7	13.4	14.7	16.1	17.7	20.7	23
07:06	90	16.2285	0.09129	-0.4359	12.7	13.4	14.7	16.2	17.8	20.8	23.1
07:07	91	16.2586	0.09254	-0.4458	12.8	13.4	14.7	16.2	17.8	20.8	23.2
07:08	92	16.2753	0.09299	-0.4528	12.8	13.4	14.7	16.2	17.8	20.8	23.3
07:09	93	16.3158	0.09311	-0.4658	12.8	13.4	14.8	16.3	17.8	20.8	23.4
07:10	94	16.3452	0.09322	-0.4789	12.8	13.4	14.8	16.3	17.8	20.9	23.5
07:11	95	16.3689	0.09355	-0.4852	12.8	13.4	14.8	16.3	17.9	20.9	23.6
08:00	96	16.4285	0.09388	-0.4951	12.9	13.4	14.8	16.4	17.9	20.9	23.7
08:01	97	16.4658	0.09399	-0.4999	12.9	13.5	14.8	16.4	18	21	23.9
08:02	98	16.4902	0.09401	-0.5044	12.9	13.5	14.8	16.4	18	21	24

08:03	99	16.5054	0.09409	-0.5124	12.9	13.5	14.8	16.5	18	21.1	24.2
08:04	100	16.5125	0.09412	-0.5214	12.9	13.5	14.9	16.5	18	21.1	24.3
08:05	101	16.668	0.09422	-0.5369	12.9	13.6	14.9	16.6	18.1	21.2	24.5
08:06	102	16.6785	0.09455	-0.5417	12.9	13.6	14.9	16.6	18.1	21.2	24.6
08:07	103	16.7075	0.09466	-0.5528	13	13.6	14.9	16.7	18.1	21.3	24.8
08:08	104	16.7285	0.09475	-0.5652	13	13.6	14.9	16.7	18.2	21.4	24.9
08:09	105	16.82358	0.09488	-0.5789	13	13.7	14.9	16.8	18.2	21.4	25.1
08:10	106	16.83568	0.09498	-0.5841	13	13.7	15	16.8	18.3	21.5	25.3
08:11	107	16.9752	0.09502	-0.5962	13	13.7	15	16.9	18.3	21.5	25.5
09:00	108	16.9963	0.09522	-0.5989	13.1	13.7	15	16.9	18.3	21.6	25.6
09:01	109	16.9996	0.09536	-0.6014	13.1	13.8	15	16.9	18.4	21.6	25.8
09:02	110	17.0456	0.09545	-0.6125	13.1	13.8	15	17	18.4	21.7	25.9
09:03	111	17.0562	0.09565	-0.6258	13.1	13.8	15.1	17	18.4	21.7	26
09:04	112	17.1185	0.09575	-0.6364	13.1	13.8	15.1	17.1	18.4	21.8	26.2
09:05	113	17.1258	0.09622	-0.6458	13.1	13.8	15.1	17.1	18.5	21.9	26.3
09:06	114	17.2685	0.09755	-0.6521	13.2	13.9	15.1	17.2	18.5	22	26.5
09:07	115	17.2285	0.09841	-0.6621	13.2	13.9	15.2	17.2	18.6	22.1	26.7
09:08	116	17.3658	0.09962	-0.6741	13.2	13.9	15.2	17.3	18.6	22.2	26.8
09:09	117	17.3952	0.10003	-0.6874	13.2	13.9	15.2	17.3	18.7	22.3	26.9
09:10	118	17.4175	0.10023	-0.6958	13.2	13.9	15.2	17.4	18.7	22.4	27
09:11	119	17.4956	0.10057	-0.6988	13.3	13.9	15.3	17.4	18.8	22.5	27.2
10:00	120	17.5458	0.10111	-0.7018	13.3	13.9	15.3	17.5	18.8	22.6	27.4
10:01	121	17.5963	0.10254	-0.7022	13.3	14	15.4	17.5	18.9	22.7	27.5
10:02	122	17.5993	0.10365	-0.7147	13.3	14	15.4	17.5	18.9	22.7	27.6
10:03	123	17.6257	0.10478	-0.7248	13.3	14	15.4	17.6	19	22.8	27.8
10:04	124	17.6658	0.10569	-0.7325	13.4	14	15.5	17.6	19	22.8	28
10:05	125	17.7175	0.10698	-0.7425	13.4	14.1	15.5	17.7	19.1	22.9	28.1
10:06	126	17.7523	0.10789	-0.7528	13.4	14.1	15.5	17.7	19.2	22.9	28.3
10:07	127	17.7785	0.10814	-0.7635	13.4	14.1	15.6	17.7	19.3	23	28.4
10:08	128	17.8201	0.10874	-0.7789	13.5	14.1	15.6	17.8	19.3	23.1	28.6
10:09	129	17.8513	0.10911	-0.7852	13.5	14.2	15.6	17.8	19.4	23.2	28.7
10:10	130	17.9074	0.10925	-0.7936	13.5	14.2	15.7	17.9	19.4	23.3	28.9
10:11	131	17.9632	0.10955	-0.8125	13.5	14.2	15.7	17.9	19.5	23.4	29
11:00	132	18.042	0.10999	-0.8258	13.6	14.3	15.7	18	19.6	23.5	29.2
11:01	133	18.067	0.11005	-0.8458	13.6	14.3	15.8	18	19.6	23.5	29.3
11:02	134	18.085	0.11009	-0.8582	13.6	14.3	15.8	18	19.7	23.6	29.5
11:03	135	18.1125	0.11022	-0.8635	13.6	14.4	15.8	18.1	19.7	23.7	29.7
11:04	136	18.1345	0.11066	-0.8952	13.7	14.4	15.9	18.1	19.8	23.7	29.8
11:05	137	18.1345	0.11089	-0.9125	13.7	14.4	15.9	18.1	19.9	23.8	29.9
11:06	138	18.22452	0.11098	-0.9365	13.7	14.5	15.9	18.2	20	23.9	30
11:07	139	18.2364	0.11123	-0.9587	13.7	14.5	16	18.2	20	24	30.2
11:08	140	18.3563	0.11129	-0.9852	13.7	14.5	16	18.3	20	24	30.4
11:09	141	18.3756	0.11132	-0.9925	13.8	14.5	16	18.3	20.1	24.1	30.5
11:10	142	18.4105	0.11149	-1.0009	13.8	14.6	16.1	18.4	20.2	24.2	30.6
11:11	143	18.4357	0.11158	-1.0099	13.8	14.6	16.1	18.4	20.3	24.3	30.7
12:00	144	18.521	0.11169	-1.0125	13.8	14.6	16.1	18.5	20.4	24.4	30.9

12:01	145	18.5732	0.11171	-1.0357	13.9	14.7	16.2	18.5	20.5	24.5	31
12:02	146	18.5993	0.11189	-1.0591	13.9	14.7	16.2	18.5	20.6	24.6	31.2
12:03	147	18.6212	0.11193	-1.0852	14	14.7	16.3	18.6	20.6	24.7	31.3
12:04	148	18.6632	0.11199	-1.0999	14	14.8	16.3	18.6	20.7	24.8	31.4
12:05	149	18.7045	0.11201	-1.1125	14	14.8	16.4	18.7	20.8	24.9	31.5
12:06	150	18.7358	0.11209	-1.1258	14	14.8	16.5	18.7	20.9	25	31.6
12:07	151	18.801	0.11233	-1.1456	14.1	14.9	16.5	18.8	20.9	25.1	31.7
12:08	152	18.9635	0.11255	-1.1658	14.1	15	16.6	18.9	21	25.2	31.8
12:09	153	19.045	0.11265	-1.1789	14.1	15	16.6	19	21.1	25.3	32
12:10	154	19.1158	0.11278	-1.1899	14.2	15	16.7	19.1	21.2	25.4	32.2
12:11	155	19.1358	0.11298	-1.1936	14.2	15.1	16.7	19.1	21.3	25.5	32.4
13:00	156	19.2258	0.11299	-1.2002	14.2	15.1	16.8	19.2	21.3	25.6	32.6
13:01	157	19.2652	0.11301	-1.2022	14.3	15.2	16.9	19.2	21.4	25.7	32.7
13:02	158	19.3275	0.11322	-1.2123	14.3	15.2	17	19.3	21.5	25.8	32.9
13:03	159	19.3358	0.11333	-1.2369	14.4	15.3	17	19.3	21.6	25.9	33
13:04	160	19.4452	0.11345	-1.2456	14.4	15.3	17.1	19.4	21.6	26	33.2
13:05	161	19.5963	0.11355	-1.2589	14.4	15.3	17.1	19.5	21.6	26.1	33.4
13:06	162	19.6052	0.11366	-1.2698	14.5	15.4	17.2	19.6	21.7	26.2	33.6
13:07	163	19.7624	0.11389	-1.2789	14.5	15.4	17.2	19.7	21.7	26.3	33.7
13:08	164	19.7963	0.11399	-1.2985	14.6	15.4	17.3	19.7	21.8	26.4	33.8
13:09	165	19.8204	0.11401	-1.3058	14.6	15.5	17.3	19.8	21.8	26.5	33.9
13:10	166	19.8602	0.11422	-1.3129	14.7	15.5	17.4	19.8	21.9	26.6	34
13:11	167	19.9304	0.11436	-1.3299	14.7	15.6	17.4	19.9	22	26.7	34.1
14:00	168	20.043	0.11444	-1.3369	14.7	15.6	17.4	20	22.1	26.8	34.2
14:01	169	20.0576	0.11455	-1.3587	14.8	15.7	17.5	20	22.2	26.9	34.3
14:02	170	20.1152	0.11465	-1.3639	14.8	15.7	17.5	20.1	22.3	27	34.4
14:03	171	20.1963	0.11478	-1.3789	14.8	15.7	17.6	20.1	22.4	27.1	34.6
14:04	172	20.2243	0.11498	-1.3985	14.9	15.8	17.6	20.2	22.5	27.2	34.8
14:05	173	20.2538	0.11499	-1.4123	14.9	15.8	17.7	20.2	22.6	27.3	34.9
14:06	174	20.3279	0.11501	-1.4369	14.9	15.9	17.8	20.3	22.7	27.4	35
14:07	175	20.3685	0.11522	-1.4759	15	15.9	17.8	20.3	22.8	27.5	35.1
14:08	176	20.4135	0.11533	-1.4999	15	16	17.9	20.4	22.8	27.6	35.2
14:09	177	20.4896	0.11587	-1.5258	15	16	17.9	20.4	22.9	27.7	35.3
14:10	178	20.5357	0.11598	-1.5456	15.1	16.1	18	20.5	23	27.8	35.4
14:11	179	20.5687	0.11601	-1.5852	15.1	16.1	18	20.5	23.1	27.9	35.6
15:00	180	20.6152	0.11622	-1.5999	15.2	16.2	18.1	20.6	23.2	28	35.7
15:01	181	20.6685	0.11636	-1.6125	15.2	16.3	18.2	20.6	23.2	28.1	35.8
15:02	182	20.6963	0.11646	-1.6456	15.2	16.3	18.3	20.6	23.3	28.2	35.9
15:03	183	20.7245	0.11656	-1.6852	15.3	16.4	18.3	20.7	23.3	28.3	36
15:04	184	20.7356	0.11678	-1.6963	15.3	16.4	18.3	20.7	23.4	28.4	36.1
15:05	185	20.7789	0.11688	-1.7003	15.3	16.5	18.4	20.7	23.4	28.5	36.2
15:06	186	20.8052	0.11699	-1.7125	15.4	16.5	18.4	20.8	23.5	28.6	36.3
15:07	187	20.8605	0.11701	-1.7009	15.4	16.5	18.5	20.8	23.5	28.7	36.3
15:08	188	20.8785	0.11711	-1.6852	15.4	16.6	18.5	20.8	23.6	28.8	36.3
15:09	189	20.9301	0.11723	-1.6521	15.5	16.6	18.6	20.9	23.7	28.9	36.4
15:10	190	21.08	0.11745	-1.6123	15.5	16.7	18.6	21	23.8	29	36.4

15:11	191	21.0963	0.11798	-1.5987	15.6	16.7	18.7	21	23.8	29.1	36.4
16:00	192	21.1342	0.11799	-1.5258	15.6	16.7	18.7	21.1	23.9	29.2	36.5
16:01	193	21.1356	0.11801	-1.4963	15.6	16.8	18.8	21.1	24	29.3	36.6
16:02	194	21.2275	0.11825	-1.4528	15.6	16.9	18.8	21.2	24.1	29.4	36.6
16:03	195	21.2369	0.11865	-1.4325	15.7	17	18.9	21.2	24.1	29.5	36.7
16:04	196	21.3356	0.11888	-1.4111	15.7	17	18.9	21.3	24.2	29.6	36.7
16:05	197	21.3563	0.11899	-1.3987	15.7	17	18.9	21.3	24.3	29.7	36.8
16:06	198	21.3756	0.11902	-1.3528	15.8	17.1	19	21.3	24.4	29.8	36.9
16:07	199	21.4205	0.11936	-1.3411	15.8	17.1	19	21.4	24.4	29.9	36.9
16:08	200	21.4356	0.11945	-1.3211	15.9	17.2	19.1	21.4	24.5	30	37
16:09	201	21.5453	0.11999	-1.3009	15.9	17.2	19.1	21.5	24.5	30.1	37
16:10	202	21.5896	0.12009	-1.2951	15.9	17.2	19.2	21.5	24.6	30.2	37
16:11	203	21.6234	0.12019	-1.2841	16	17.2	19.2	21.6	24.6	30.3	37
17:00	204	21.6863	0.12036	-1.2211	16	17.2	19.3	21.6	24.7	30.4	37
17:01	205	21.6932	0.12099	-1.2022	16	17.3	19.3	21.6	24.7	30.4	37.1
17:02	206	21.69921	0.12111	-1.1528	16.1	17.3	19.4	21.6	24.8	30.5	37.1
17:03	207	21.7025	0.12112	-1.1321	16.1	17.3	19.4	21.7	24.8	30.6	37.1
17:04	208	21.7325	0.12236	-1.1009	16.1	17.4	19.5	21.7	24.9	30.6	37.1
17:05	209	21.7685	0.12325	-1.0899	16.1	17.4	19.5	21.7	24.9	30.7	37.2
17:06	210	21.8985	0.12456	-1.0528	16.1	17.4	19.5	21.8	25	30.7	37.2
17:07	211	21.8996	0.12526	-1.0235	16.1	17.4	19.5	21.8	25.1	30.8	37.2
17:08	212	21.8999	0.12599	-1.0009	16.1	17.4	19.6	21.8	25.1	30.8	37.2
17:09	213	21.90257	0.12601	-0.9963	16.2	17.5	19.6	21.9	25.2	30.9	37.2
17:10	214	21.92145	0.12636	-0.9852	16.2	17.5	19.6	21.9	25.2	30.9	37.2
17:11	215	22.0258	0.12689	-0.9753	16.2	17.5	19.7	22	25.2	31	37.2
18:00	216	22.3548	0.12699	-0.9654	16.2	17.5	19.7	22	25.3	31	37.2
18:01	217	22.1125	0.12714	-0.9521	16.2	17.6	19.7	22.1	25.3	31.1	37.3
18:02	218	22.1355	0.12752	-0.9421	16.3	17.6	19.7	22.1	25.4	31.1	37.3
18:03	219	22.2357	0.12798	-0.9325	16.3	17.6	19.8	22.2	25.4	31.2	37.3
18:04	220	22.2685	0.12799	-0.9254	16.3	17.7	19.8	22.2	25.4	31.2	37.3
18:05	221	22.2963	0.12801	-0.9125	16.3	17.7	19.8	22.2	25.5	31.3	37.3
18:06	222	22.3235	0.12822	-0.9111	16.3	17.7	19.9	22.3	25.5	31.3	37.3
18:07	223	22.3657	0.12833	-0.8936	16.3	17.8	19.9	22.3	25.6	31.4	37.3
18:08	224	22.3769	0.12855	-0.8852	16.3	17.8	19.9	22.3	25.6	31.4	37.3
18:09	225	22.4025	0.12866	-0.8745	16.3	17.8	20	22.4	25.6	31.4	37.3
18:10	226	22.4357	0.12865	-0.8625	16.3	17.8	20	22.4	25.7	31.4	37.3
18:11	227	22.4568	0.12898	-0.8528	16.3	17.8	20	22.4	25.7	31.4	37.3
19:00	228	22.4963	0.12899	-0.8421	16.3	17.8	20	22.4	25.7	31.4	37.3

Table (6): shows Egyptian L, M and S parameters and Z score for BMI for age for girls from 5 years to 19 years

BMI-for-age GIRLS<br>Egyptian Z-score 5 Years to 19 Years											
Y:M	M	Mean	S	L	-3SD	-2SD	-1SD	Median	1SD	2SD	3SD
05:01	61	15.5985	0.08852	-0.7965	12	13	14.5	15.5	17.5	20.6	21.7
05:02	62	15.5756	0.08963	-0.8569	12	13	14.5	15.5	17.5	20.7	21.8
05:03	63	15.5685	0.08999	-0.8899	12	13	14.5	15.5	17.5	20.8	21.9
05:04	64	15.544	0.09009	-0.8999	12	13	14.5	15.5	17.5	20.8	22
05:05	65	15.5356	0.09011	-0.9002	12	13	14.5	15.5	17.5	20.9	22
05:06	66	15.5305	0.09123	-0.9016	12	13	14.5	15.5	17.6	20.9	22.1
05:07	67	15.5012	0.09222	-0.9111	12	13	14.5	15.5	17.6	21	22.2
05:08	68	15.4756	0.09299	-0.9569	12	13	14.5	15.5	17.6	21	22.3
05:09	69	15.4358	0.09369	-0.9889	12	13	14.5	15.5	17.6	21.1	22.4
05:10	70	15.6420	0.09456	-1.0258	12	13	14.5	15.6	17.6	21.2	22.5
05:11	71	15.6589	0.09588	-1.0369	12	13	14.5	15.6	17.6	21.3	22.6
06:00	72	15.6689	0.09623	-1.0458	12	13	14.5	15.6	17.7	21.3	22.7
06:01	73	15.686	0.09789	-1.0789	12	13	14.5	15.6	17.7	21.3	22.7
06:02	74	15.696	0.09825	-1.0899	12	13	14.5	15.6	17.7	21.3	22.8
06:03	75	15.704	0.09963	-1.0936	12	13	14.5	15.7	17.7	21.3	22.9
06:04	76	15.7357	0.09999	-1.1022	12	13	14.5	15.7	17.7	21.3	23
06:05	77	15.7583	0.10015	-1.1055	12	13	14.5	15.7	17.7	21.4	23.1
06:06	78	15.7862	0.10049	-1.1125	12	13	14.5	15.7	17.8	21.4	23.2
06:07	79	15.7963	0.10109	-1.1222	12	13	14.5	15.7	17.8	21.4	23.3
06:08	80	15.8042	0.10199	-1.1325	12	13	14.5	15.8	17.8	21.4	23.3
06:09	81	15.8358	0.10212	-1.1369	12	13	14.5	15.8	17.8	21.5	23.4
06:10	82	15.8458	0.10222	-1.1399	12	13	14.5	15.8	17.8	21.5	23.4
06:11	83	15.7687	0.10236	-1.1411	12	13	14.5	15.8	17.9	21.5	23.5
07:00	84	15.8358	0.10288	-1.1502	12	13	14.5	15.8	17.9	21.6	23.6
07:01	85	15.8785	0.10299	-1.1602	12.1	13	14.6	15.8	17.9	21.6	23.7
07:02	86	15.8962	0.10311	-1.1632	12.1	13	14.6	15.9	17.9	21.7	23.9
07:03	87	15.994	0.10333	-1.1706	12.1	13	14.6	15.9	18	21.8	24
07:04	88	15.999	0.10365	-1.1709	12.1	13	14.6	15.9	18	21.8	24.2
07:05	89	16.014	0.10399	-1.1836	12.1	13	14.6	16	18	21.9	24.4
07:06	90	16.045	0.10411	-1.1936	12.1	13	14.7	16	18.1	21.9	24.6
07:07	91	16.068	0.10454	-1.1989	12.1	13.1	14.7	16.1	18.1	22	24.7
07:08	92	16.115	0.10499	-1.2009	12.1	13.1	14.7	16.1	18.1	22	24.8
07:09	93	16.1358	0.10501	-1.2055	12.1	13.1	14.8	16.1	18.2	22.1	25
07:10	94	16.2258	0.10522	-1.2123	12.1	13.1	14.8	16.2	18.2	22.2	25.1
07:11	95	16.2456	0.10533	-1.2254	12.1	13.1	14.8	16.2	18.2	22.3	25.2
08:00	96	16.3358	0.10555	-1.2299	12.1	13.1	14.9	16.3	18.3	22.4	25.4
08:01	97	16.3456	0.10599	-1.2311	12.2	13.2	14.9	16.3	18.3	22.5	25.5
08:02	98	16.4752	0.10612	-1.2345	12.2	13.2	14.9	16.4	18.3	22.6	25.7

08:03	99	16.4962	0.10632	-1.2398	12.2	13.2	14.9	16.4	18.4	22.7	25.9
08:04	100	16.5254	0.10657	-1.2417	12.2	13.2	14.9	16.5	18.4	22.7	26
08:05	101	16.5532	0.10666	-1.2587	12.3	13.2	15	16.5	18.4	22.8	26.1
08:06	102	16.6014	0.10699	-1.2599	12.3	13.3	15	16.6	18.5	22.9	26.2
08:07	103	16.6856	0.10711	-1.2602	12.3	13.3	15	16.6	18.5	23	26.3
08:08	104	16.7245	0.10755	-1.2613	12.3	13.3	15.1	16.7	18.6	23	26.5
08:09	105	16.7625	0.10766	-1.2687	12.3	13.3	15.1	16.7	18.6	23.1	26.6
08:10	106	16.812	0.10777	-1.2699	12.3	13.3	15.1	16.8	18.7	23.1	26.8
08:11	107	16.8358	0.10799	-1.2709	12.3	13.3	15.2	16.8	18.7	23.2	26.9
09:00	108	16.8752	0.10801	-1.2711	12.4	13.4	15.2	16.9	18.8	23.2	27
09:01	109	16.9015	0.10932	-1.2789	12.4	13.4	15.2	16.9	18.8	23.2	27.2
09:02	110	16.9358	0.11002	-1.2799	12.4	13.4	15.2	17	18.9	23.3	27.3
09:03	111	17.075	0.11125	-1.2802	12.5	13.4	15.3	17.1	19	23.3	27.5
09:04	112	17.0956	0.11236	-1.2825	12.5	13.5	15.3	17.1	19	23.4	27.6
09:05	113	17.1158	0.11369	-1.2855	12.5	13.5	15.3	17.2	19.1	23.5	27.8
09:06	114	17.2357	0.11456	-1.2888	12.6	13.5	15.4	17.3	19.2	23.6	27.9
09:07	115	17.3102	0.11569	-1.2899	12.6	13.6	15.4	17.3	19.3	23.7	28
09:08	116	17.4357	0.11698	-1.2905	12.6	13.6	15.4	17.4	19.4	23.8	28.2
09:09	117	17.4852	0.11754	-1.2946	12.6	13.6	15.5	17.4	19.4	23.9	28.4
09:10	118	17.5421	0.11852	-1.2988	12.7	13.7	15.5	17.5	19.4	24	28.6
09:11	119	17.5965	0.11963	-1.2999	12.7	13.7	15.5	17.5	19.5	24.1	28.7
10:00	120	17.654	0.11999	-1.3005	12.7	13.7	15.5	17.6	19.5	24.2	28.8
10:01	121	17.635	0.12009	-1.3009	12.8	13.8	15.5	17.6	19.5	24.2	28.9
10:02	122	17.7752	0.12125	-1.3041	12.8	13.8	15.5	17.7	19.6	24.3	29
10:03	123	17.8245	0.12258	-1.3069	12.9	13.8	15.6	17.8	19.6	24.4	29.2
10:04	124	17.885	0.12369	-1.3099	12.9	13.9	15.6	17.8	19.7	24.5	29.3
10:05	125	17.9175	0.12456	-1.3148	12.9	14	15.6	17.9	19.8	24.5	29.5
10:06	126	17.9358	0.12569	-1.3189	13	14	15.6	17.9	19.9	24.6	29.7
10:07	127	17.99912	0.12698	-1.3201	13	14	15.7	18	20	24.7	29.9
10:08	128	18.028	0.12789	-1.3222	13	14	15.7	18	20.1	24.8	30.1
10:09	129	18.1258	0.12852	-1.3299	13.1	14.1	15.7	18.1	20.2	24.9	30.3
10:10	130	18.2352	0.12963	-1.3302	13.1	14.1	15.8	18.2	20.3	25	30.5
10:11	131	18.2856	0.12987	-1.3311	13.1	14.1	15.8	18.2	20.4	25.1	30.6
11:00	132	18.3358	0.13006	-1.3333	13.2	14.2	15.8	18.3	20.5	25.2	30.7
11:01	133	18.3697	0.13022	-1.3458	13.2	14.2	15.9	18.3	20.5	25.3	30.9
11:02	134	18.3965	0.13055	-1.3569	13.3	14.3	15.9	18.4	20.6	25.3	31
11:03	135	18.4258	0.13066	-1.3698	13.3	14.3	16	18.5	20.7	25.4	31.1
11:04	136	18.5635	0.13099	-1.3789	13.4	14.4	16.1	18.6	20.8	25.5	31.2
11:05	137	18.6752	0.13101	-1.3852	13.5	14.5	16.1	18.7	20.9	25.6	31.4
11:06	138	18.7963	0.13111	-1.3914	13.5	14.5	16.2	18.8	21	25.7	31.6
11:07	139	18.8258	0.13122	-1.4008	13.6	14.6	16.3	18.8	21	25.8	31.7
11:08	140	18.9452	0.13133	-1.4123	13.6	14.6	16.4	18.9	21.1	25.8	31.8
11:09	141	19.0586	0.13144	-1.4254	13.7	14.7	16.5	19	21.2	25.9	32
11:10	142	19.0863	0.13155	-1.4369	13.8	14.7	16.5	19.1	21.2	26	32.2
11:11	143	19.2158	0.13166	-1.4411	13.9	14.8	16.6	19.2	21.3	26.1	32.3
12:00	144	19.2752	0.13177	-1.4399	14	14.9	16.6	19.2	21.4	26.2	32.4

12:01	145	19.3345	0.13189	-1.4302	14	15	16.7	19.3	21.5	26.3	32.5
12:02	146	19.3752	0.13196	-1.4287	14.1	15.1	16.7	19.3	21.6	26.4	32.6
12:03	147	19.4756	0.13201	-1.4211	14.1	15.1	16.8	19.4	21.6	26.5	32.7
12:04	148	19.5258	0.13222	-1.4205	14.2	15.2	16.8	19.5	21.7	26.6	32.8
12:05	149	19.6658	0.13236	-1.4066	14.2	15.2	16.9	19.6	21.8	26.7	32.9
12:06	150	19.7258	0.13245	-1.4033	14.3	15.3	16.9	19.7	21.8	26.8	33
12:07	151	19.7685	0.13255	-1.3987	14.3	15.4	17	19.7	21.9	26.9	33.2
12:08	152	19.8258	0.13266	-1.3903	14.4	15.4	17	19.8	22	27	33.4
12:09	153	19.8685	0.13278	-1.3888	14.4	15.4	17.1	19.8	22	27.1	33.6
12:10	154	19.9375	0.13289	-1.3814	14.5	15.5	17.1	19.9	22.1	27.2	33.8
12:11	155	19.9658	0.13333	-1.3802	14.5	15.5	17.2	19.9	22.2	27.3	33.9
13:00	156	20.0158	0.13345	-1.3798	14.5	15.5	17.2	20	22.3	27.4	34
13:01	157	20.0452	0.13355	-1.3789	14.5	15.5	17.3	20.1	22.4	27.4	34.1
13:02	158	20.2257	0.13366	-1.3711	14.6	15.6	17.3	20.2	22.5	27.5	34.2
13:03	159	20.3358	0.13378	-1.3705	14.6	15.6	17.3	20.3	22.5	27.6	34.3
13:04	160	20.4425	0.13388	-1.3687	14.7	15.7	17.4	20.4	22.6	27.7	34.4
13:05	161	20.4961	0.13399	-1.3674	14.8	15.8	17.4	20.4	22.7	27.7	34.5
13:06	162	20.3258	0.13401	-1.3611	14.8	15.8	17.5	20.4	22.8	27.8	34.6
13:07	163	20.5354	0.13411	-1.3609	14.9	15.9	17.5	20.5	22.8	27.9	34.7
13:08	164	20.6752	0.13422	-1.3587	14.9	15.9	17.5	20.6	22.9	28	34.8
13:09	165	20.7015	0.13444	-1.3578	15	16	17.6	20.7	23	28.1	34.9
13:10	166	20.8752	0.13454	-1.3533	15.1	16	17.6	20.8	23.1	28.2	35
13:11	167	20.9346	0.13455	-1.3511	15.1	16	17.7	20.9	23.2	28.3	35.1
14:00	168	20.9892	0.13466	-1.3505	15.1	16.1	17.7	20.9	23.3	28.4	35.2
14:01	169	21.0157	0.13477	-1.3485	15.1	16.1	17.8	21	23.3	28.5	35.3
14:02	170	21.0358	0.13488	-1.3357	15.2	16.2	17.8	21	23.4	28.6	35.4
14:03	171	21.1125	0.13499	-1.3258	15.2	16.2	17.9	21.1	23.5	28.7	35.5
14:04	172	21.2345	0.13501	-1.3222	15.2	16.2	17.9	21.2	23.6	28.7	35.6
14:05	173	21.2962	0.13511	-1.3199	15.2	16.2	18	21.2	23.6	28.8	35.7
14:06	174	21.3204	0.13522	-1.3155	15.2	16.2	18	21.3	23.7	28.9	35.7
14:07	175	21.3820	0.13533	-1.3142	15.2	16.2	18.1	21.3	23.7	29	35.8
14:08	176	21.4123	0.13544	-1.3101	15.3	16.3	18.1	21.4	23.8	29	35.8
14:09	177	21.47125	0.13555	-1.3009	15.3	16.3	18.2	21.4	23.8	29.1	35.9
14:10	178	21.5264	0.13565	-1.2999	15.3	16.3	18.2	21.5	23.9	29.2	35.9
14:11	179	21.5852	0.13575	-1.2951	15.3	16.3	18.3	21.5	23.9	29.3	36
15:00	180	21.6486	0.13585	-1.2911	15.3	16.4	18.3	21.6	24	29.4	36
15:01	181	21.5382	0.13599	-1.2896	15.3	16.4	18.4	21.6	24	29.5	36.1
15:02	182	21.719	0.13601	-1.2888	15.3	16.4	18.4	21.7	24.1	29.6	36.1
15:03	183	21.7204	0.13622	-1.2855	15.3	16.4	18.4	21.7	24.1	29.6	36.2
15:04	184	21.8587	0.13633	-1.2843	15.3	16.4	18.4	21.8	24.2	29.7	36.2
15:05	185	21.8375	0.13644	-1.2801	15.3	16.5	18.5	21.8	24.2	29.8	36.3
15:06	186	21.9352	0.13654	-1.2799	15.3	16.5	18.5	21.9	24.3	29.9	36.4
15:07	187	21.9963	0.13636	-1.2788	15.3	16.6	18.5	21.9	24.3	30	36.4
15:08	188	22.0178	0.13646	-1.2763	15.3	16.7	18.6	22	24.4	30	36.5
15:09	189	21.9258	0.13656	-1.2751	15.4	16.7	18.6	22	24.5	30.1	36.5
15:10	190	22.1358	0.13666	-1.2741	15.4	16.8	18.7	22.1	24.5	30.2	36.5

15:11	191	22.2346	0.13676	-1.2733	15.4	16.8	18.7	22.2	24.6	30.2	36.6
16:00	192	22.3852	0.13686	-1.2707	15.4	16.9	18.8	22.3	24.7	30.3	36.6
16:01	193	22.3992	0.13696	-1.2632	15.4	16.9	18.8	22.3	24.7	30.3	36.6
16:02	194	22.415	0.13701	-1.2606	15.4	16.9	18.8	22.4	24.8	30.3	36.6
16:03	195	22.4752	0.13711	-1.2599	15.4	16.9	18.8	22.4	24.8	30.3	36.6
16:04	196	22.5589	0.13722	-1.2684	15.4	16.9	18.8	22.5	24.9	30.4	36.7
16:05	197	22.5962	0.13733	-1.2598	15.4	16.9	18.9	22.5	24.9	30.4	36.7
16:06	198	22.5996	0.13745	-1.2541	15.4	17	18.9	22.5	25	30.4	36.7
16:07	199	22.6357	0.13755	-1.2458	15.4	17	18.9	22.6	25	30.4	36.7
16:08	200	22.6657	0.13765	-1.2436	15.4	17	18.9	22.6	25.1	30.5	36.7
16:09	201	22.7052	0.13775	-1.2369	15.5	17	18.9	22.7	25.1	30.5	36.7
16:10	202	22.73457	0.13785	-1.2258	15.5	17	18.9	22.7	25.2	30.5	36.7
16:11	203	22.8573	0.13795	-1.2125	15.5	17	18.9	22.8	25.2	30.5	36.7
17:00	204	22.8758	0.13799	-1.2099	15.5	17.1	19	22.8	25.3	30.6	36.7
17:01	205	22.8893	0.13801	-1.2006	15.5	17.1	19	22.8	25.3	30.6	36.8
17:02	206	22.8925	0.13811	-1.1952	15.5	17.1	19	22.9	25.3	30.6	36.8
17:03	207	22.9012	0.13823	-1.1847	15.5	17.1	19	22.9	25.3	30.6	36.8
17:04	208	22.8325	0.13833	-1.1741	15.5	17.1	19	22.9	25.4	30.6	36.8
17:05	209	23.0258	0.13844	-1.1632	15.5	17.1	19	23	25.4	30.7	36.8
17:06	210	23.0462	0.13855	-1.1528	15.5	17.1	19.1	23	25.4	30.7	36.8
17:07	211	23.0785	0.13865	-1.1478	15.5	17.1	19.1	23	25.5	30.7	36.8
17:08	212	23.0953	0.13875	-1.1321	15.5	17.1	19.1	23.1	25.5	30.7	36.8
17:09	213	23.0992	0.13885	-1.1254	15.5	17.1	19.1	23.1	25.5	30.7	36.8
17:10	214	23.0999	0.13898	-1.1156	15.5	17.1	19.1	23.1	25.6	30.7	36.8
17:11	215	23.1125	0.13902	-1.1099	15.5	17.1	19.2	23.2	25.6	30.8	36.8
18:00	216	23.1235	0.13911	-1.0958	15.5	17.1	19.2	23.2	25.6	30.8	36.8
18:01	217	23.1423	0.13923	-1.0852	15.5	17.2	19.2	23.2	25.6	30.8	36.8
18:02	218	23.2652	0.13932	-1.0789	15.5	17.2	19.2	23.2	25.6	30.8	36.8
18:03	219	23.3011	0.13955	-1.0652	15.5	17.2	19.2	23.3	25.6	30.8	36.8
18:04	220	23.335	0.13966	-1.0002	15.5	17.2	19.2	23.3	25.7	30.8	36.8
18:05	221	23.3754	0.13975	-0.0952	15.5	17.2	19.3	23.3	25.7	30.8	36.7
18:06	222	23.4246	0.13985	-0.0965	15.5	17.2	19.3	23.4	25.7	30.8	36.7
18:07	223	23.4485	0.13999	-0.0912	15.5	17.2	19.3	23.4	25.7	30.8	36.7
18:08	224	23.4679	0.14009	-0.0865	15.5	17.2	19.3	23.4	25.7	30.8	36.7
18:09	225	23.5210	0.14101	-0.0861	15.5	17.2	19.3	23.5	25.8	30.8	36.7
18:10	226	23.5632	0.14222	-0.0852	15.5	17.2	19.3	23.5	25.8	30.9	36.7
18:11	227	23.5785	0.14369	-0.0874	15.5	17.2	19.3	23.5	25.8	30.9	36.7
19:00	228	23.5354	0.14465	-0.0723	15.5	17.2	19.3	23.5	25.8	30.9	36.7
